# Hydrogel fibers for wearable sensors and soft actuators

**DOI:** 10.1016/j.isci.2023.106796

**Published:** 2023-05-03

**Authors:** Jiaxuan Du, Qing Ma, Binghao Wang, Litao Sun, Limei Liu

**Affiliations:** 1 School of Electronic Science & Engineering, Southeast University, Nanjing, Jiangsu 210096, China; 2 College of Mechanical Engineering, Yangzhou University, Yangzhou, Jiangsu 225127, China

**Keywords:** Sensor, Devices, Biomedical materials

## Abstract

Owing to superior softness, wetness, responsiveness, and biocompatibility, bulk hydrogels are being intensively investigated for versatile functions in devices and machines including sensors, actuators, optics, and coatings. The one-dimensional (1D) hydrogel fibers possess the metrics from both the hydrogel materials and structural topology, endowing them with extraordinary mechanical, sensing, breathable and weavable properties. As no comprehensive review has been reported for this nascent field, this article aims to provide an overview of hydrogel fibers for soft electronics and actuators. We first introduce the basic properties and measurement methods of hydrogel fibers, including mechanical, electrical, adhesive, and biocompatible properties. Then, typical manufacturing methods for 1D hydrogel fibers and fibrous films are discussed. Next, the recent progress of wearable sensors (e.g., strain, temperature, pH, and humidity) and actuators made from hydrogel fibers is discussed. We conclude with future perspectives on next-generation hydrogel fibers and the remaining challenges. The development of hydrogel fibers will not only provide an unparalleled one-dimensional characteristic, but also translate fundamental understanding of hydrogels into new application boundaries.

## Graphical abstract

**Figure:**
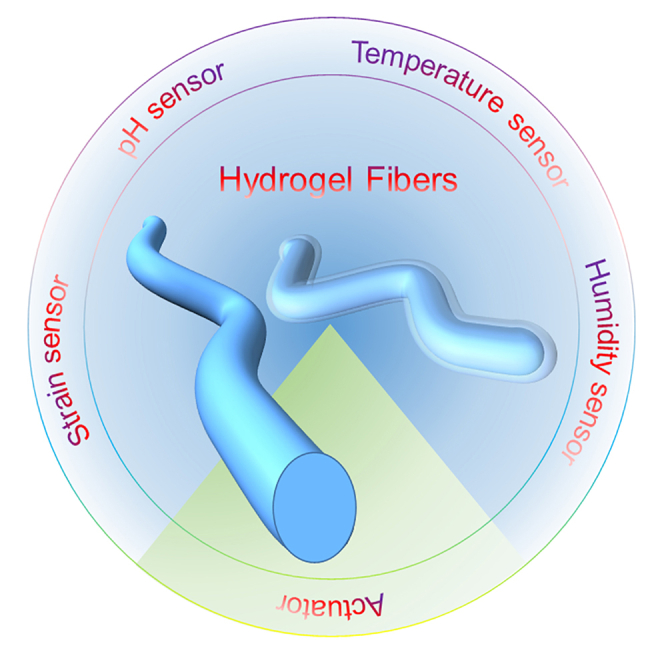

## Introduction

Hydrogels are viscoelastic materials consisting of a three-dimensional network of chemically or physically cross-linked hydrophilic polymers that have the ability to absorb and retain large amounts of water.^1^^,^^2^^,^^3^^,^^4^ Hydrogels have the advantages of excellent stretchability, flexibility, self-healing and biocompatibility, and by introducing electrical conductors (e.g., graphene, carbon nanotubes) and ions (e.g., NaCl, KCl), they are endowed with certain electrical/ion conduction properties, making them ideal materials for flexible sensors.^5^^,^^6^^,^^7^^,^^8^^,^^9^^,^^10^ The sensing function is achieved by measuring the changes in electrical signals such as current, voltage, resistance, and capacitance of conductive hydrogels subjected to external stimuli (e.g., pressure, temperature, humidity, and pH).^11^^,^^12^^,^^13^^,^^14^ Compared with sensors based on other materials (e.g., silicon, metals, carbon materials, metal oxides, and conducting polymers), conductive hydrogels have obvious advantages in terms of stretchability, flexibility, adhesion, ease of preparation, and biocompatibility, which have been widely reported for the applications in the fields of wearable electronics, bioelectronics, and soft robotics.^15^^,^^16^^,^^17^^,^^18^

Bulk hydrogels have been extensively studied because of their simplicity of preparation; however, these conductive hydrogels are large and have an isotropic homogeneous structure, so the response rate and sensitivity are limited. In recent years, a wide variety of hydrogel forms (e.g., columns, spheres, films, fibers, and granules) have been developed.^19^^,^^20^^,^^21^^,^^22^^,^^23^ These hydrogel forms are available in sizes ranging from nanometers to millimeters to meet different application scenarios. Among them, hydrogel fibers have been reported for bio-scaffolds, wound dressings and surgical suture owing to its quasi-one-dimensional structure, good breathability and weaveability.^24^^,^^25^^,^^26^^,^^27^^,^^28^^,^^29^^,^^30^ Likewise, conductive hydrogel fibers (CHFs), when used as sensors, exhibit anisotropic responsiveness, enhanced specific surface area and better air permeability compared to bulk hydrogels. Also, CHFs can be woven and non-woven into hydrogel textile with 3D structures, which exhibit good conformability. Furthermore, CHF-based devices can act as soft actuators that respond well to external stimuli.^31^

Despite the great promise and recent advances in hydrogel fibers, to the best of our knowledge, there is no systematic discussion on hydrogel fibers for wearable sensors and actuators. The literature on bulk hydrogels for tissue engineering, drug delivery, soft machines, and human-machine interfaces has been extensively reviewed, but existing reviews usually do not account for unique 1D hydrogel fibers nor do they provide the fabrication and applications of hydrogel fibers.^32^^,^^33^^,^^34^^,^^35^ Such a systematic discussion is central for the future development of this nascent yet impactful field.

**Figure 1:**
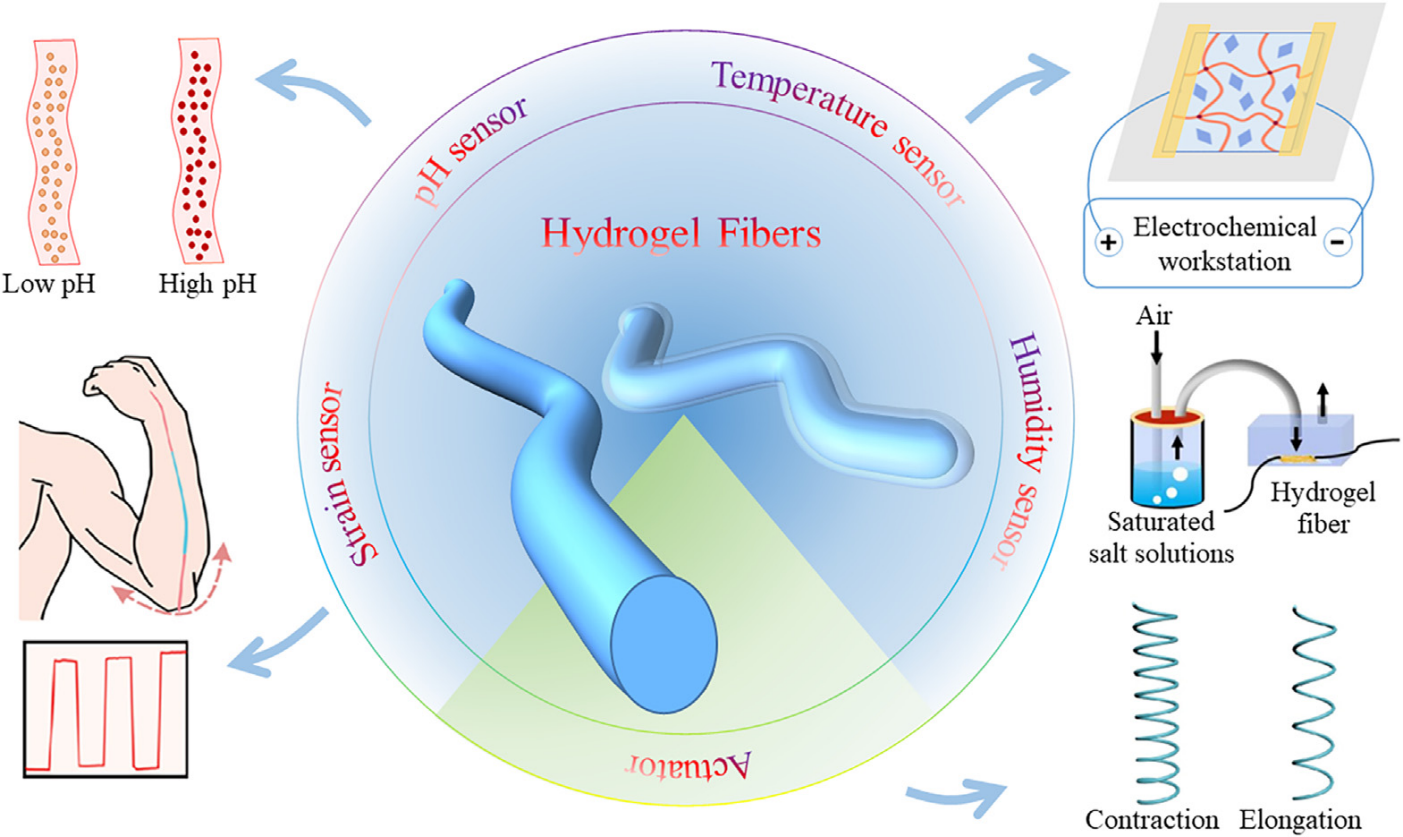
Schematic diagram of the application of hydrogel fibers in the field of sensors and soft actuators Reproduced with permission.^124^ Copyright 2020, American Chemical Society. Reproduced with permission.^103^ Copyright 2020, WILEY-VCH.

In this Review, we first summarize the basic properties of hydrogel fibers that are closely related to flexible electronics. Then we summarize the methodologies for fabricating hydrogel fibers with diameter varying from nanometers to millimeters. In the following part, which constitutes the largest section, recent studies on hydrogel fibers for flexible electronics are summarized, with emphasis on applications for strain, temperature, PH and humidity sensors and actuators. Figure 1 provides applications of hydrogel fibers in the field of sensors and soft actuators and Table 1 summarizes the materials, design strategies, and performance of the sensors and actuators relevant to this paper. We conclude with future perspectives on next-generation hydrogel fibers and the remaining challenges and opportunities.

**Table 1:** Summary of materials, design strategies and performance of the electronic devices relevant to this article

Application	Material	Method	Diameter (μm)	Mechanical properties (strength/elongation)	Conductivity (S m^−1^)	Working range^a^	Year^Ref^
Strain sensor	Alginate and PAAm	Template	420	80 kPa/730%	N/A	0-120%	2016^91^
	Sodium polyacrylate and SWCNT	Wet spinning	900	1.2 MPa/2630%	88.7	0-1000%	2022^22^
	PAAm	Template	1200	2000%	0.16	N/A	2019^92^
	Alginate and PEGDA	Wet spinning	450	400%	0.765	50-300%	2020^93^
	PEDOT:PSS and PVA	Template	1000	13 MPa/519%	0.163	0.01–130%	2022^73^
	SA/PANI/rGO	Wet spinning	180	10.36 MPa/154%	0.5	10-50%	2022^94^
	KGM/KC/PANI	Electrospinning	0.25	239.26 kPa/340.69%	0.7261	N/A	2022^95^
Temperature sensor	PVA and HEC	Template	800	2.86 MPa/400%	5.77	28-45°C	2022^98^
	Chitosan and polypyrrole	Wet spinning	244	872 MPa/2%	310	10-40°C	2011^99^
pH-sensitive sensor	H-PAN and SP	Wet spinning	90	N/A	N/A	3–10.5	2008^100^
	Alginate and glycerol	Microfluidic spinning	200–1000	N/A	N/A	5.2–9	2016^101^
	NIPAm and SA	Template	220	N/A	N/A	3–11	2018^102^
Humidity sensor	P(AAm-co-AA)/Fe(III)	Draw-spinning	85	N/A	0.0042	10-90 RH%	2020^103^
	Agarose/SMF/PCF	Template	153	N/A	N/A	10-90 RH%	2014^104^
Actuator	TPU/P(NIPAM-ABP)	Electrospinning	501	N/A	N/A	N/A	2015^114^
	GO and alginate	Wet spinning	500	N/A	N/A	N/A	2020^115^
	PAA and PCL	Electrospinning	1.28	N/A	N/A	N/A	2022^118^

a The unit of working range for strain sensor is strain. The unit of working range for temperature sensor is °C. The unit of working range for humidity sensor is RH%.

## Properties of hydrogel fibers

Hydrogel fibers are increasingly used in wearable electronics, requiring hydrogel fibers with mechanical durability, electrical conductivity, certain stretchability, adhesion, and biocompatibility etc. Good mechanical properties with certain stretchability and adhesion, endow the devices with excellent conformability and minimized motion artifacts during movements. The desirable electrical properties and water-retention ability ensure excellent device performance and long-term operation stability. Furthermore, a good biocompatibility of the hydrogel fibers is a prerequisite when used for wearable electronics.

### Mechanical properties

**Figure 2:**
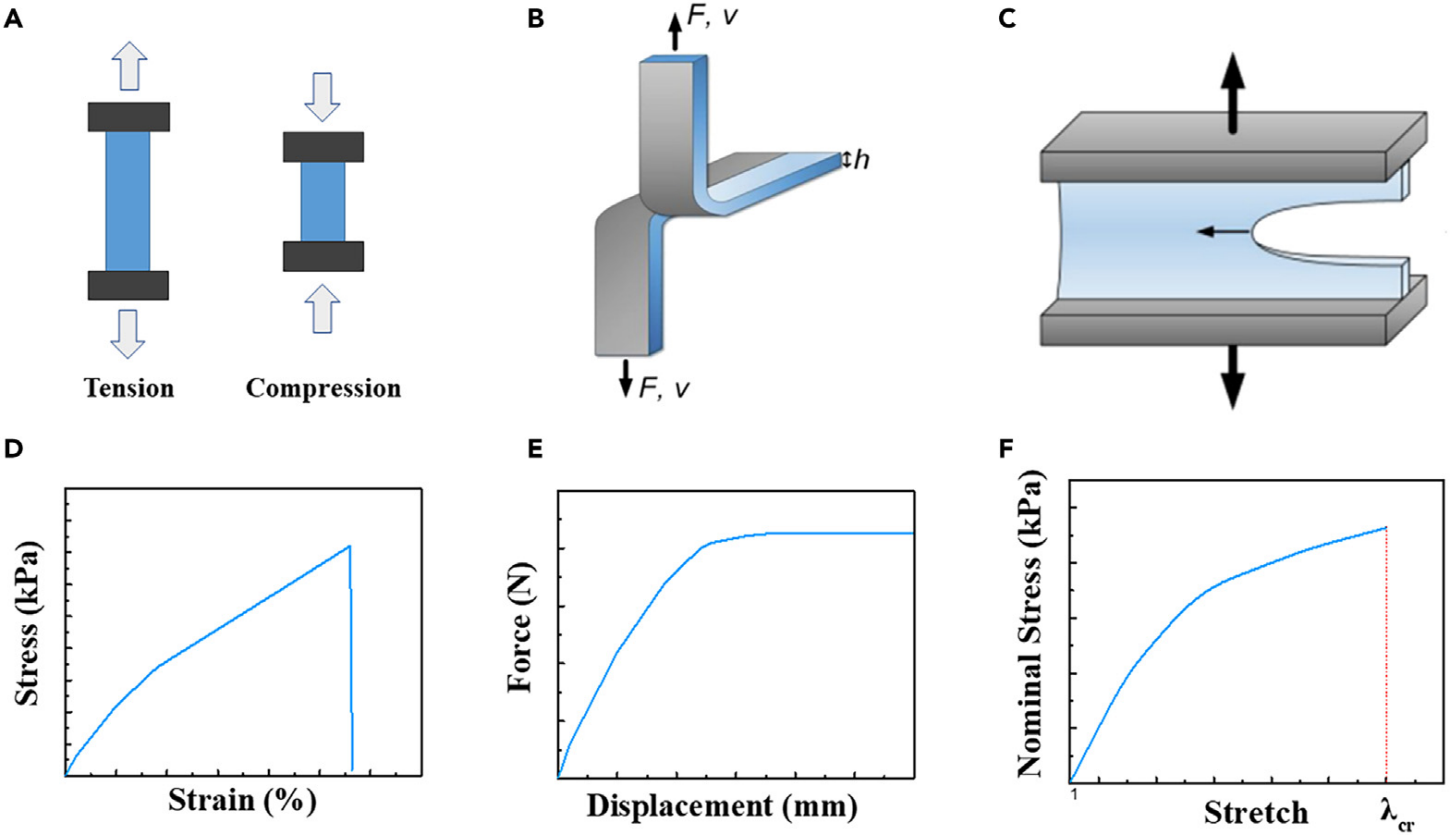
Mechanical property test (A) Experimental diagram of applying tensile load to hydrogel to measure mechanical properties. (B) Schematic diagram of tear test. (C) Schematic diagram of the single-notch method. (D) Diagram of tensile test results. (E) Force-displacement curves of tear tests. (F) Diagram of fracture toughness test results. (B and C) Reproduced with permission.^38^ Copyright 2018, Elsevier Masson SAS.

Hydrogel fibers require a certain mechanical strength for practical use.^36^ Several important parameters, including tensile/compression strength, elastic modulus, elongation, tear strength, fracture toughness and fatigue threshold have been proposed.^37^ Figure 2A shows the schematic of tensile test, which involves applying a longitudinal tensile load to the hydrogel fiber sample using a universal testing machine under fixed temperature and humidity conditions. From the stress-strain curve (Figure 2D), tensile strength, elastic modulus at different strains, and elongation can be obtained. For those tough hydrogel fiber membranes, tear strength is used to characterize the tear resistance of the samples.^38^ The setup is some to tensile test, whereas the test sample has an intentional cut (Figure 2B). Thus, tear resistance (with a unit of kN/m) is then calculated by dividing the force applied by the thickness of the sample.

The fracture toughness and fatigue threshold of hydrogel fiber membranes is usually determined using the single-notch method (Figure 2C).^39^ To measure fracture toughness, a notch was cut from the edge of the specimen using a razor blade. The stress-tensile curves of the same specimens with and without notches were then measured. The fracture toughness of the hydrogel is given by Equation 1:

(Equation 1) $$\Gamma =W\left(\lambda_{cr}\right)H$$

*W (λ*_*cr*_*)* is the area below the nominal stress-stretch curve of the unslotted sample, where *λ*_*cr*_ is the critical stretch of the slotted sample, and *H* is the initial length of the sample. To measure the fatigue threshold of hydrogels, the maximum tensile cyclic load was applied to the hydrogel samples with cracks, and the curve of crack extension dc/dN was obtained for each cycle.^40^ The applied energy release rate *G* in the notched sample under the N_th_ cycle of applied stretch *λ*_*A*_ can be calculated as

(Equation 2) $$G\left(\lambda_{A}\right)=2k\left(\lambda_{A}\right)c\left(N\right)W\left(\lambda_{A}\right)$$

where $k=1/\sqrt{\lambda_{A}}$*, c* is the crack length without deformation, and *W* is the strain energy density stored in the unslotted sample. Fatigue threshold is obtained by linearly extrapolating the curve of dc/dN versus G to the intercept of the transverse coordinate.^41^

### Electrical property

**Figure 3:**
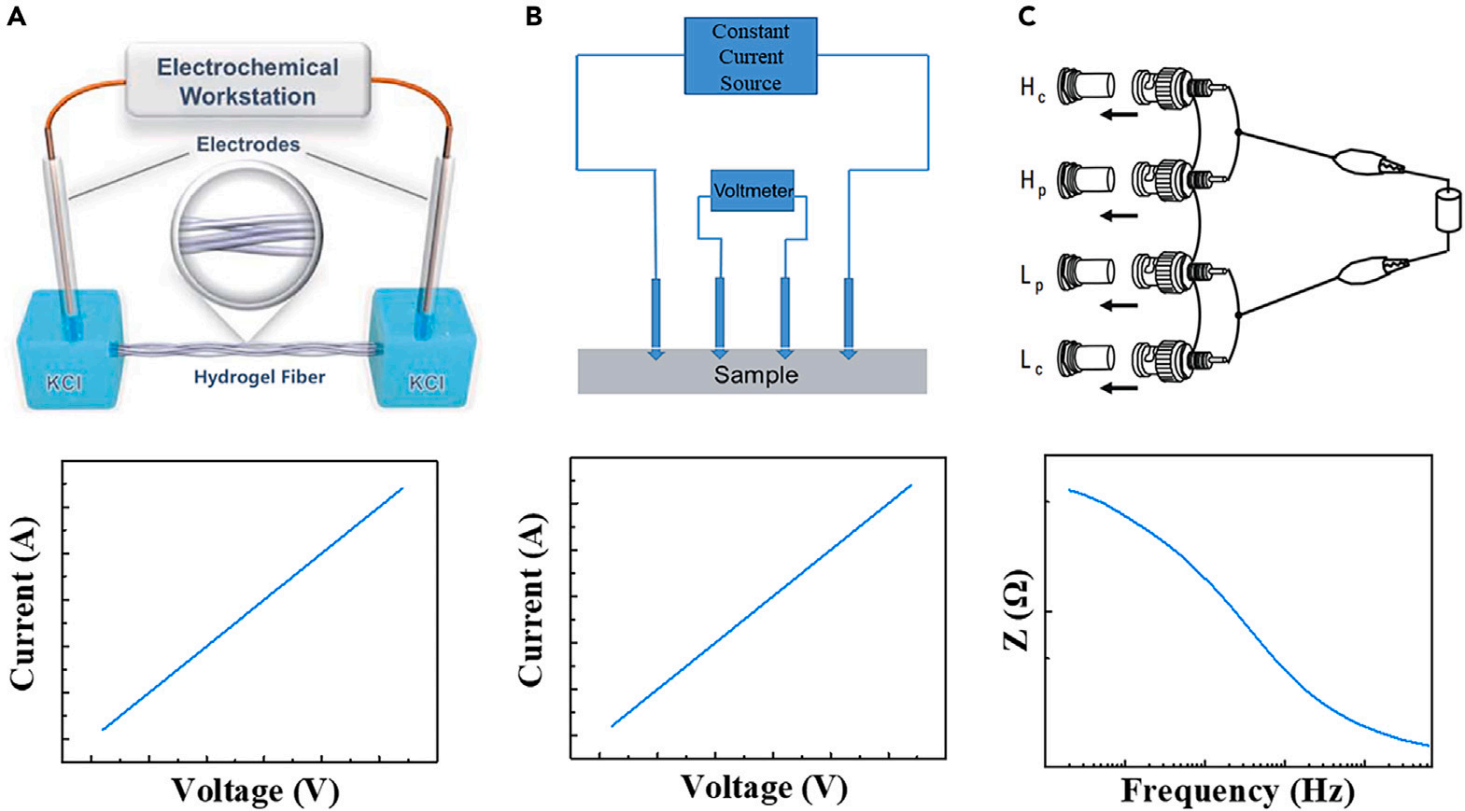
Electrical conductivity test (A) Schematic diagram and result diagram of measuring ionic conductivity of hydrogel fiber by electrochemical workstation. Reproduced with permission.^142^ Copyright 2021, The Royal Society of Chemistry 2021. (B) Schematic diagram and result diagram of measuring of hydrogel fiber membrane electrical conductivity by four-probe method. (C) Schematic diagram and result diagram of electrochemical impedance test.

The electrical conductivity of hydrogels is originated from the charge movements in the three-dimensional network, which can be classified as electron-conducting, ion-conducting, and mixed ion-electron conducting.^42^ A bunch of conductive fillers, such as metal nanoparticles, carbon nanotubes, graphene, MXene, metal salts, ionic liquids, conductive polymers (such as polyaniline (PANI), poly (3,4-ethylenedioxythiophene): poly (styrene sulfonate) (PEDOT: PSS), polythiophene (PTh) and polypyrrole (PPy) have been incorporated into hydrogels.^43^^,^^44^^,^^45^^,^^46^^,^^47^^,^^48^^,^^49^^,^^50^^,^^51^ However, most of hydrogels still exhibit relatively low conductivity (<1 S cm^−1^) owing to the existence of large amount of water (>80 wt %). The resistance of CHFs is generally calculated by the law of resistance:

(Equation 3) $$R=\rho \frac{L}{S}$$

where *ρ* is resistivity, *L* is length, *S* is cross sectional area. As shown in Figure 3 A, the conductivity of the hydrogel fiber can be measured through the electrochemical workstation. Also, we can obtain sheet resistance of the CHFs by using four-probe test method. The four-probe test uses four equally spaced probes to contact the samples’ surface (Figure 3B), and the potential difference between the two measurement points inside is obtained by introducing an external current, which ultimately measures the conductivity of the material. When the length and width of the measured sample are much larger than the probe spacing, the formula for calculating the sheet resistance of the material is give in Equation 4:

(Equation 4) $$R_{s}=\frac{\pi }{\ln\left(2\right)}\cdot \frac{V}{I}=4.53236\frac{V}{I}$$

where *V* is the change in voltage measured between the inner probes, and *I* is the current applied between the outer probes.

When CHFs are used as bioelectrodes, the electrical impendence between CHFs and tissue is also an important parameter. Electrochemical impedance spectroscopy is one of the most important tools to evaluate the electrochemical properties. The AC impedance method uses a small amplitude AC voltage or current to disturb the electrode material and then generates a sinusoidal AC signal to implement the test electrochemical properties. An instrument is usually equipped with four connectors, namely high potential (*H*_p_), high current (*H*_c_), low potential (*L*_p_), low current (*L*_c_), as measurement terminals. The measured sample is connected to the terminals by the two-terminal configuration, as shown in Figure 3C.

### Adhesion properties

**Figure 4:**
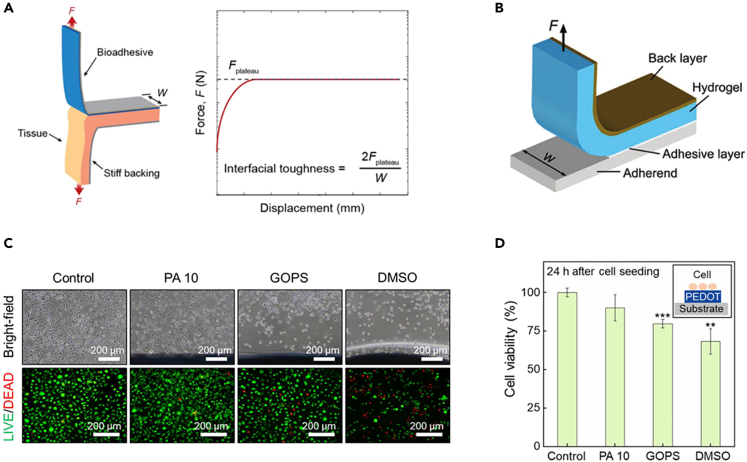
Adhesion and biocompatibility property test (A) Schematic diagram of hydrogel adhesion test. Reproduced with permission.^52^ Copyright 2020, National Academy of Sciences. (B) Schematic diagram of 90° peel test. Reproduced with permission.^143^ Copyright 2019, WILEY-VCH. (C) Morphological images of biocompatibility measured by MTT method. (D) Graph of cell viability results. (C and D) Reproduced under the terms of the Creative Commons Attribution License (http://creativecommons.org/licenses/by/4.0/).^144^ Copyright 2022, the Authors, some rights reserved. PA 10: PEDOT:PPS and AuNP; GOPS: (3-glycidyloxypropyl) trimethoxysilane.

To study adhesion properties, bioadhesion and interfacial toughness between hydrogels and different material interfaces are often measured based on a standard test for tissue adhesives, namely the 180° peel test.^52^ Peel tests measure adhesion toughness, which means the amount of energy needed to advance separation per unit area. The mechanical test setup for interfacial toughness measurement based on the standard 180° peel test is shown in Figure 4A, and the interfacial adhesion toughness (also called peel strength) can be calculated by dividing twice the plateau force (*F*) by the width of the sample (*W*), as shown in Equation 5.

(Equation 5) $$Adhesion\;toughness=\frac{{2F}_{plateau}}{W}$$

Another method of measuring adhesion is the 90° peel test, which is generally used to measure the adhesion properties of hydrogels to rigid materials. By fixing one end of the hydrogel in the fixture of the instrument, the hydrogel is peeled vertically from the surface of the material and the adhered material is moved horizontally, thus ensuring that the hydrogel remains at a 90° angle to the material (Figure 4B). The adhesion toughness (= *F*/*W*) can be calculated by dividing the force by the sample width.

### Water retention stability

Hydrogel materials have physiological and mechanical properties similar to those of human skin and consist mainly of a network of biomacromolecules permeated with water. The water content in fresh CHFs is usually more than 90 wt %, which ensures them with excellent softness, adhesion, ion and/or electron transport, etc. Nevertheless, the dehydration of CHFs is inevitability in ambient as water inevitably evaporates, these hydrogels are easy to dry, rendering them unworkable.^53^ Therefore, it is important to improve the water retention stability of CHFs.

The water retention stability of hydrogels can be measured by the following method. The hydrogel samples were first soaked in distilled water to reach equilibrium dissolution. Next, the excess water was dried and placed in a constant temperature and humidity chamber for a sustained period of time until a constant weight was reached, and the hydrogels were weighed at predetermined intervals and the weight changes of the samples were recorded.

### Biocompatibility

When used for wearable electronics, the biocompatibility of CHFs becomes predominantly important as it is highly related to human health.^54^ Thus, the monomers/polymers selected for hydrogel synthesis, the initiators, crosslinkers, and conductive fillers used, and the choice of polymerization method all have a certain degree of effect on the final cytotoxicity.

To test biocompatibility, the cellular activity of cells against hydrogels is usually assessed using the colorimetric MTT (4,5-dimethylthiazol-2-yl)-2,5-diphenyltetrazolium bromide) method or the Live/Dead staining method.^55^ Specifically, the hydrogel with 10% the size of the cell culture vessel was first placed at the bottom, certain number of cells were then seeded into the vessel with culture medium. After one day, MTT solution (5 mg/mL) reagent was added and incubated for another 4 h. Dimethylsulfoxide (DMSO) solution was added to each well and finally the absorbance value of each well was measured using a microplate reader, which can indirectly reflect the number of live cells.^56^ In addition, the Live/Dead Viability/Cytotoxicity Kit is a quick and easy two-color assay to determine viability of cells in a population. It includes calcein acetoxymethyl ester and propidium iodide (Calcein AM/PI) reagents, which stain live and dead cells, respectively. The live cells (green) or dead cells (red) can be observed under a fluorescent microscope (Figures 4C and 4D).

## Fabrication of hydrogel fibers

Unlike traditional polymer fibers, hydrogel fibers are more challenging to fabricate due to limited material choices such as polyvinyl alcohol (PVA), PEDOT: PSS, hyaluronic acid (HA) and alginate.^57^^,^^58^^,^^59^^,^^60^ Currently, electrospinning, wet spinning, dynamic cross-linked spinning, template method, 3D printing and microfluidic spinning have been developed to fabricate hydrogel fibers with diameters ranging from nanometers to millimeters.

### Electrospinning

Electrospinning is a continuous process of forming fibers under high voltage after the polymer solution is ejected through a needle.^61^ Specifically, the prepared polymer solution is fed through a needle with a diameter range of millimeter, which is driven by an electromechanical injector system. A high voltage is applied to the solution to form dangling droplets and the surface of the liquid is twisted into a conical shape called a Taylor cone. Once the voltage exceeds a critical value, the electrostatic force overcomes the solution surface tension and a steady jet of liquid is ejected from the tip of the cone. As the jet passes through the air, the solvent evaporates, leaving behind ultra-fine polymer fibers that collect on a grounded collector. The jet typically follows a curved or spiral trajectory created by the interaction between the external electric field and the charge on the jet surface. The collector is usually tubular, rotating not only along its own axis, but also moving from side to side. Depending on the solution viscosity and feed speed, it usually takes tens of minutes to hours to obtain the optimal thickness of fibrous film that can be detached from the collector. Electrospinning is a simple but efficient way to fabricate with diameter range from tens of nanometers to several microns. Thus, such hydrogel fibers are typically used in the form of fibrous membranes.

**Figure 5:**
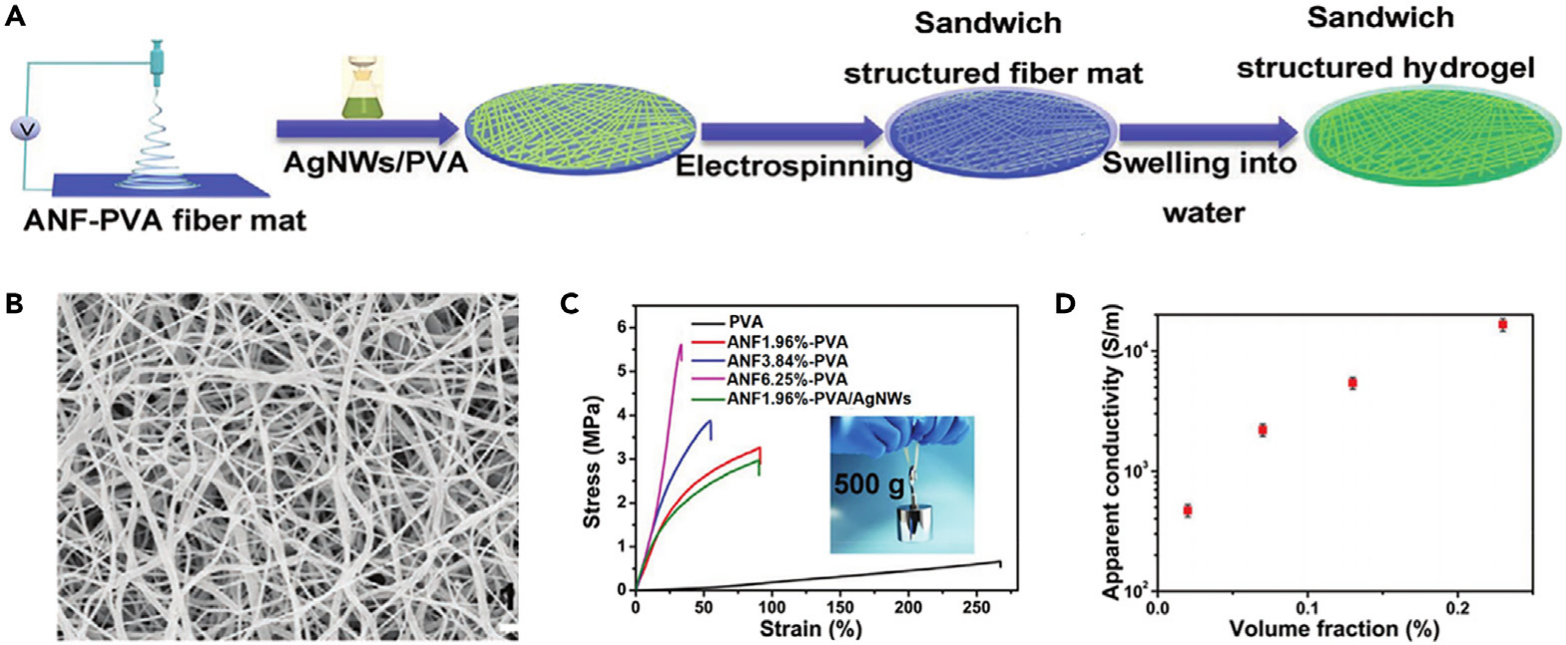
Hydrogel fibers prepared by electrospinning (A) Schematic diagram of the manufacturing procedure of hydrogel fiber mats prepared by electrospinning method. (B) SEM micrograph of ANF-PVA fiber mat. (C) Tensile stress-strain curves. (D) Sheet resistance of ANF-PVA/AgNWs hydrogels with different AgNWs contents. (A–D) Reproduced with permission.^62^ Copyright 2021, Wiley-VCH.

Zhou et al. prepared a hybrid hydrogel fiber mat based on a sandwich structure of aramid nanofibers (ANFs) reinforced polyvinyl alcohol (PVA) hydrogel layer and silver nanowires (AgNWs)/PVA layer by electrospinning combined with vacuum-assisted filtration, as shown in Figure 5A.^62^ The 10 wt % PVA and 2 wt % ANF solutions were mixed together to obtain homogeneous solutions. Fiber mats were obtained by electrospinning of ANF-PVA solutions under 24 kV applied voltage and 68°C, followed by vacuum drying at 100°C for 12 h. ANF-PVA hydrogels were prepared by soaking ANF-PVA fiber mats in deionized water for 24 h. The sandwich structured conductive ANF-PVA/AgNWs hybrid hydrogel was then prepared by depositing AgNWs/PVA suspension by vacuum filtration on top of the ANF-PVA fiber mat and then electrospinning another layer of ANF-PVA fiber mat on it. The diameter of fibers in ANF-PVA fiber mat was about 77 nm (Figure 5B). The mechanical properties of the ANF-PVA hydrogel were significantly improved with a small amount of ANF (1.96 wt %) in the hydrogel, with a strength of 3.3 MPa (Figure 5C). When the content of AgNWs in the mixed hydrogel fibers varied, the conductivity of the prepared hydrogels ranged from 470 to 1.66 × 10^4^ S m^−1^ (Figure 5D), which was higher than that of most conductive hydrogels.

### Wet spinning

**Figure 6:**
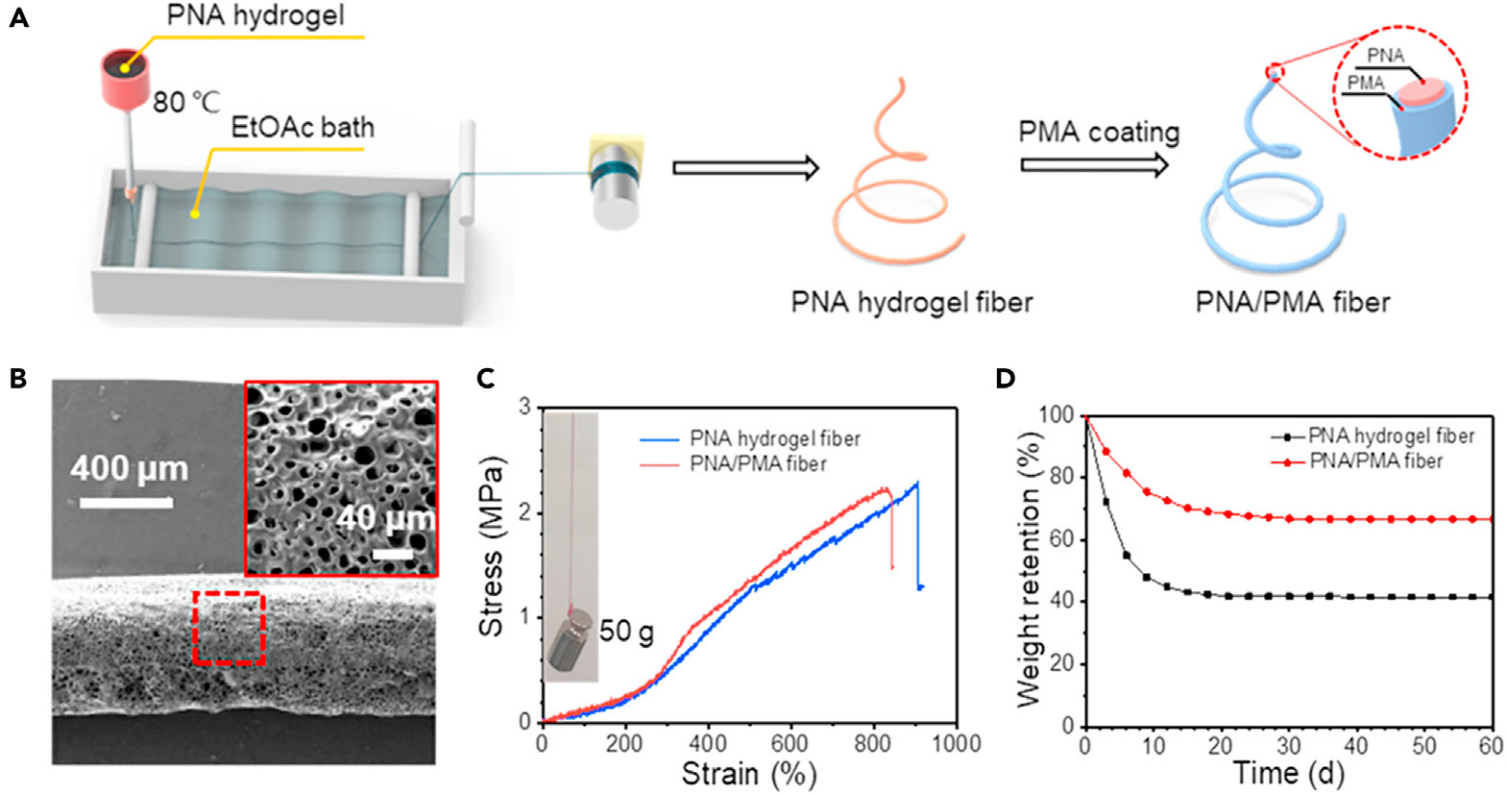
Hydrogel fibers prepared by wet spinning (A) Schematic illustration of the preparation procedures of PNA hydrogel fibers and PNA/PMA fibers. (B) SEM image of a PNA hydrogel fiber. (C) Stress-strain curves of PNA and PNA/PMA hydrogel fibers. (D) Weight retention of PNA and PNA/PMA fibers with time at room temperature and 40 RH%. Reproduced with permission.^64^ Copyright 2020, Elsevier Ltd.

Wet spinning is a widely reported method for the preparation of hydrogel fibers, in which the polymer is dissolved in a solvent and the solution is extruded into a chemical bath. The equipment required for wet spinning consists of a supply tank for the spinning solution, syringe pump, filter, spinneret, and coagulating bath. The hydrogel fibers prepared by wet spinning are generally tens to hundreds of microns in diameter. Wang et al. successfully prepared hydrogel fibers of sodium alginate/gelatin blend by wet spinning process using Ca^2+^ cross-linked sodium alginate and oxidized starch cross-linked gelatin as raw materials.^63^ A series of 4 wt % sodium alginate/gelatin aqueous solutions were prepared at room temperature for 4 h and oxidized starch was added at 5 wt % of gelatins. The solution was then added to the spinning bath and degassed under vacuum for 12 h before extrusion from a 900-hole (diameter:70 μm) spinneret into a coagulation bath containing 5 wt % aqueous calcium chloride solution. The tensile strength and elongation of the sodium alginate/gelatin blended hydrogel fibers initially increased with the amount of gelatin, reaching a maximum of 1.29 cN/dtex and 4.41%, respectively, when the gelatin content is 16.67 wt %. Further increasing the gelatin content decreases both the tensile strength and elongation. Shuai et al. developed a continuous wet spinning method to fabricate stretchable and self-healing hydrogel fibers (Figures 6A and 6B).^64^ Hydrogels were prepared by photoinitiated co-polymerization of N-acryloylglycinamide (NAGA) and acrylamide (AAm) monomers in 5 wt % LiCl aqueous solution, and then heated to make the hydrogels into viscosol state for wet spinning. After that, poly (NAGA-co-AAm) (PNA) hydrogel fiber was coated with a thin layer of poly (methyl acrylate) (PMA) by dip coating. The resulting physically cross-linked hydrogel fiber exhibits excellent tensile performances (Figure 6C), high conductivity and excellent resistance to water evaporation (Figure 6D).

**Figure 7:**
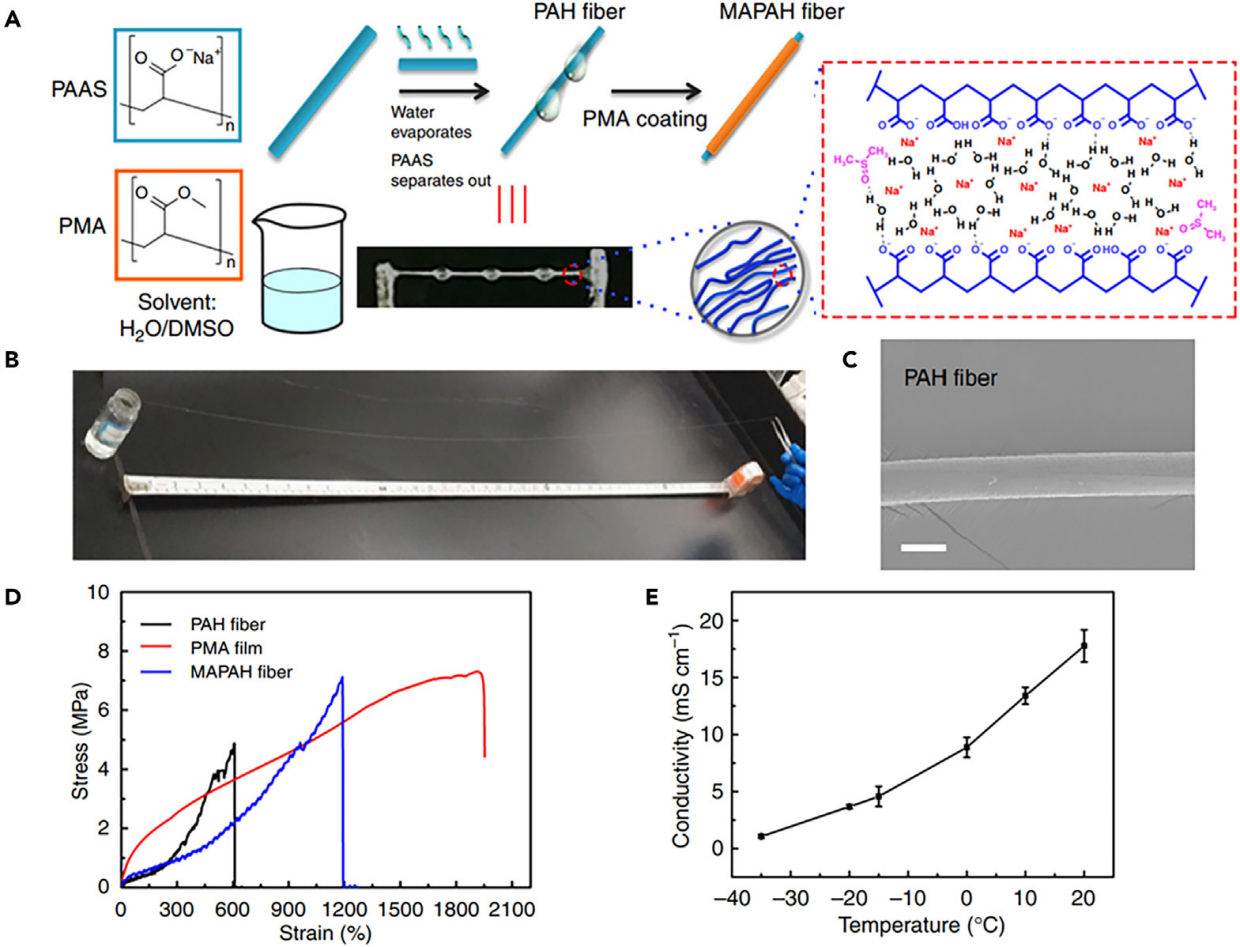
Hydrogel fibers prepared by draw spinning (A) Schematic diagram of MAPAH fibers prepared by traction stretching filament formation method. (B) Photograph of a PAH fiber (1.1 m). (C) SEM image of surface of a PAH fiber. (D) Stress–strain profiles of a PMA film, PAH, and MAPAH fibers. (E) Conductivity of MAPAH fibers in the temperature range of −35-25°C. (A–E) Reproduced under the terms of the CC-BY Creative Commons Attribution 4.0 International License (http://creativecommons.org/licenses/by/4.0/).^65^ Copyright 2018.

Spider silk is a fiber of protein, produced by a gland in the spider’s abdomen. When the spider needs silk, the liquefied protein in the gland passes through a canal filling with acid, then the liquid protein changes into fiber structure. Inspired by this, Zhao et al. prepared CHFs with ordered molecular chains and reversible chain arrangement using high molecular weight polyelectrolytes by a simple spinning method.^65^ Noting that in aqueous solution, the anionic carboxylic acid groups on the sodium polyacrylate (PAAS) chain repel each other by double-layer forces, which results in the polymer chain adopting an expanded rigid rod-like conformation, the authors added PAAS to a mixture of water and the poor solvent dimethylsulfoxide (DMSO), and then hydrogel fibers were obtained by spinning from the gel-like PAAS solution (Figure 7A). Long and uniform filaments can be readily drawn from the solution. During such process, water evaporation from the PAAS filaments in air enriches the DMSO in the filaments, which triggers a quick phase transition to form PAAS hydrogel (PAH) fibers (Figure 7B), the SEM images of which is shown in Figure 7C. The PAH fiber exhibits a diameter of ∼100 μm with a unique beads-on-a-string structure. After encapsulating with a thin layer of polymethyl acrylate, the resulting core–shell fibers exhibit a unique combination of high tensile strength (5.6 MPa) and stretchability (1200%), fast resilience (<30 s), good electrical conductivity (2 Sm^−1^), and great anti-freezing property (Figures 7D and 7E). The hydrogel fibers exhibit the excellent mechanical properties, and even exceed that of the natural spider silk.^66^^,^^67^

### Dynamic cross-linked spinning

**Figure 8:**
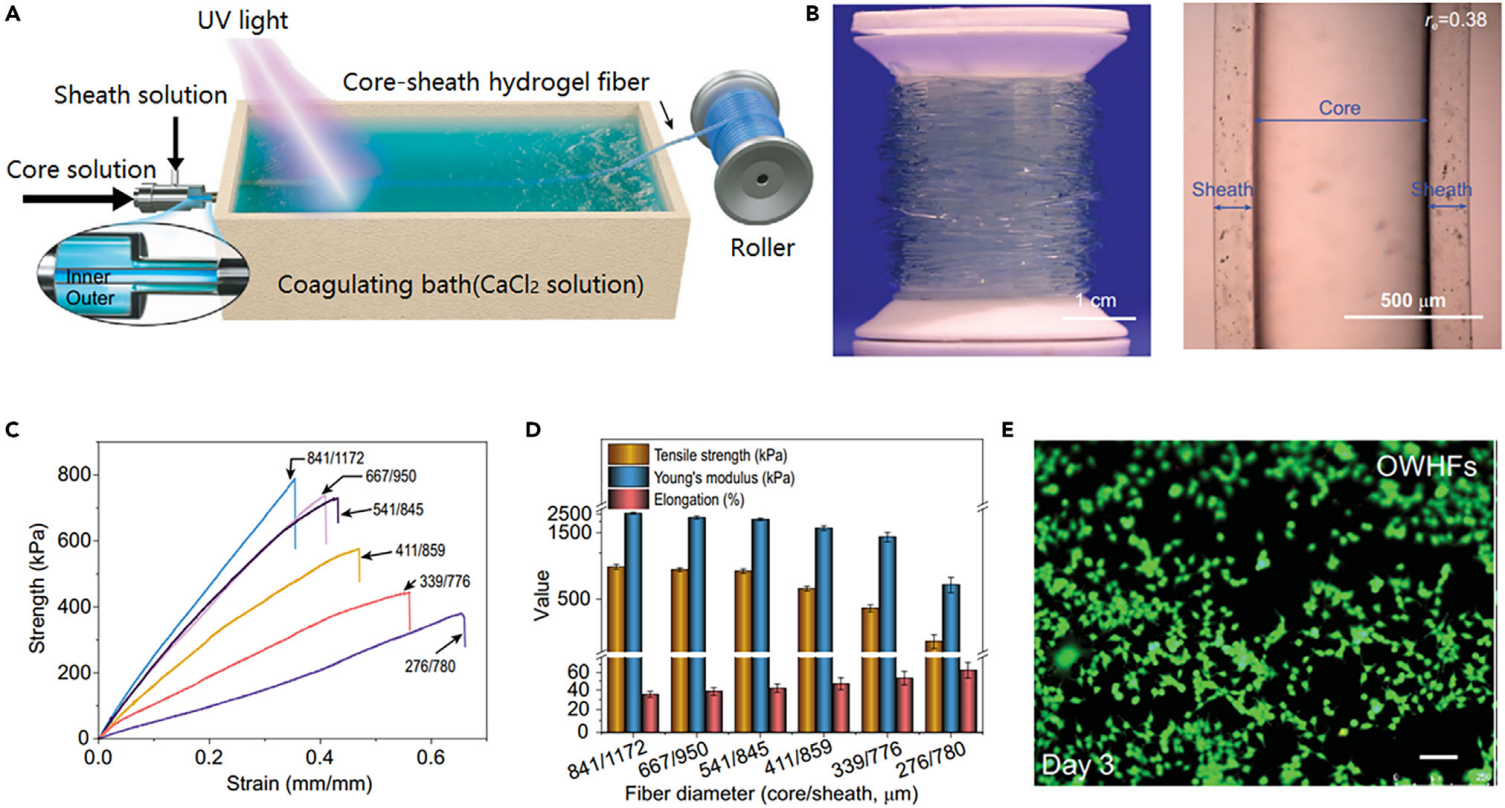
Hydrogel fibers prepared by dynamic cross-linked spinning (A) Schematic illustration of the integrated dynamic spinning apparatus. (B) Photograph and side-view optical image of a rolled core-sheath hydrogel fiber. (C) Tensile stress-strain curves. (D) Strength, elongation and modulus of the optical waveguide hydrogel fiber (OWHF) dependent on the fiber diameters. (E) Live/dead assay of cells on OWHF at day 3. (A–E) Reproduced under the terms of the Creative Commons Attribution License (http://creativecommons.org/licenses/by/4.0/).^72^ Copyright 2020.

The dynamic cross-linked spinning (DCS) method skillfully synchronizes fiber formation and gelation through rapid photopolymerization.^68^ The hydrogel fiber formation mechanism of the DCS method is based on chemical reaction, which is different form the conventional wet spinning, where the hydrogel fibers are typically formed by non-solvent phase separation and crosslinking, etc.^69^ Zhu’s group developed a novel DCS method for the facile preparation of size-controlled hydrogel fibers from polyethylene glycol diacrylate (PEGDA) oligomers without the use of microfluidic chips.^70^ The diameter of hydrogel fibers can be precisely adjusted by varying the conditions of hydrogel concentration, extrusion rate and winding speed. In a following work, the authors selected N, N-Dimethylacrylamide (DMAA) and oligo (ethylene glycol) methacrylate (OEGMA) as the spinning monomers and adjusted the diameter to the range of 194–338 μm by the DCS method.^71^ The obtained hydrogel fibers exhibit enhanced mechanical properties and high Young’s modulus (16.14 MPa). This spinning strategy will show great prospects in designing novel low-dimensional materials, such as artificial muscles and skin. In the same year, they reported an integrated optically triggered dynamic cross-linked spinning concept capable of continuous production of core-shell hydrogel fibers with tunable fiber diameters (Figure 8A).^72^ The core spinning solution is a mixture of PEGDA and AAm, and the sheath spinning solution is Na-alginate aqueous solution. As shown in Figure 8B, the hydrogel fiber up to 10 m in length was synthesized and collected on a bobbin. The wet spinning synthesis process and fabrication process were optimized by rational design of core/sheath material interfacial compatibility, optical transparency, refractive index and spinning solution viscosity. The resulting hydrogel fibers exhibited desirable low optical attenuation, tissue-like Young’s modulus (<2.60 MPa) (Figures 8C and 8D) and excellent biocompatibility (Figure 8E).

### Template method

The template method is also a simple and effective way to prepare hydrogel fibers, though the scalable fabrication of thin fibers is challenge. For instance, the PEDOT:PSS/PVA hydrogel fiber was successfully fabricated by injecting a homogeneous mixture into a glass capillary with an inner diameter of ∼1 mm, followed by removing the blue PEDOT:PSS@PVA hydrogel fiber after five cycles of repeated freezing and thawing at −20°C.^73^ The ultimate tensile strength of the PEDOT:PSS@PVA-5 fiber can reach 13.8 MPa with 519.9% of elongation at break. The diameter of the prepared hydrogel fiber was 240 μm and the hydrogel fibers had outstanding water retention property and low-temperature resistance because of the addition of glycerol. Kim et al. prepared hydrogel fibers with interpenetrating polymer network (IPN) consisting of polyacrylamide (PAAm) and alginate using the template method. Compared with PAAm hydrogel fiber, the mechanical properties of the composite hydrogels were significantly improved.^74^ The tensile strength and elongation of PAAm/alginate hydrogel fiber are 408 kPa and 426% respectively, much higher than those of PAAm hydrogel fiber.

**Figure 9:**
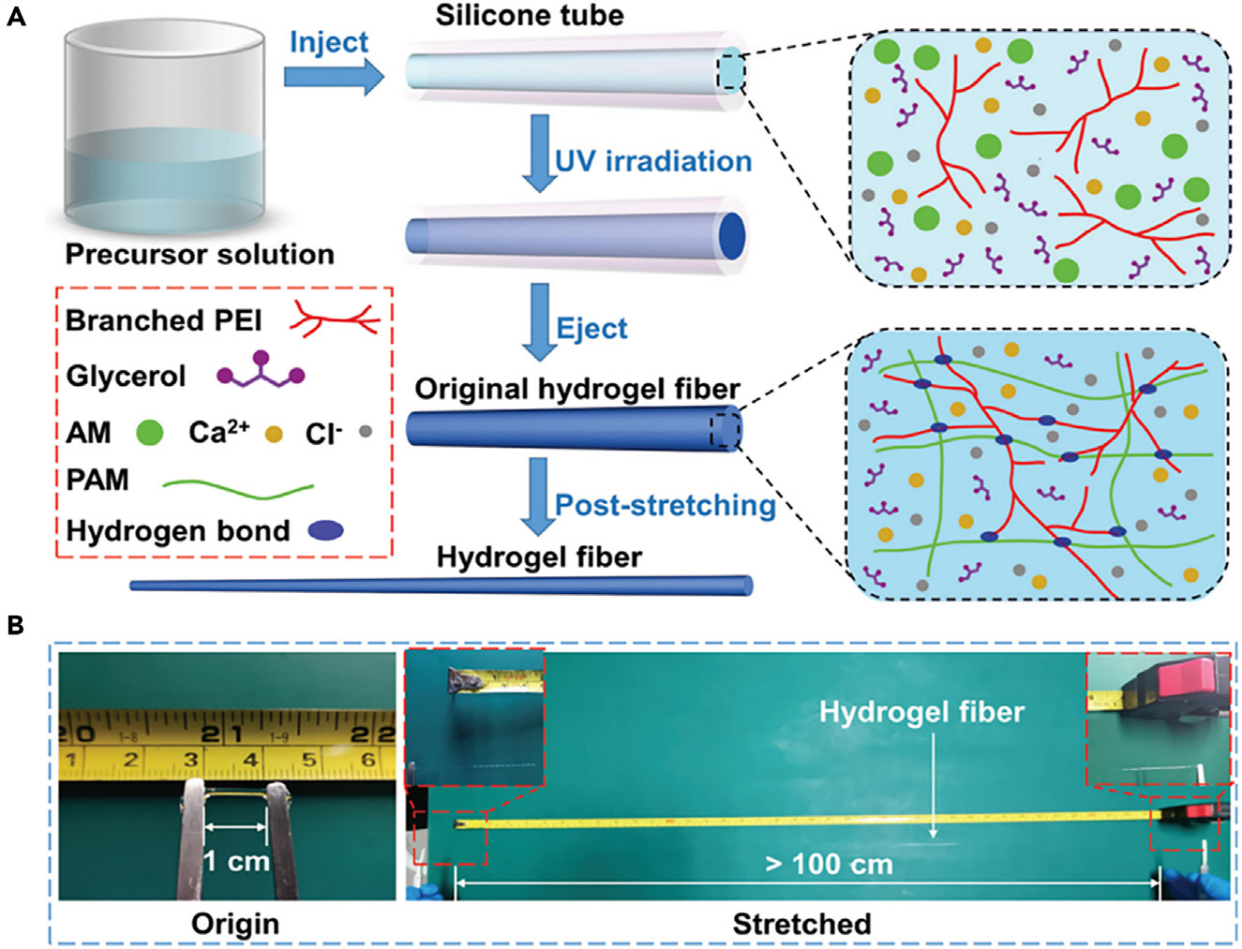
Hydrogel fibers prepared by template method (A) Schematic diagram of the preparation of hydrogel fibers by the template method. (B) Photos of the prepared hydrogel fiber stretched 100 times. (A-B) Reproduced with permission.^75^ Copyright 2021, The Royal Society of Chemistry.

Xu et al. prepared adhesive functional hydrogel fibers by post-stretching the pre-cured PAAm-polyethyleneimine-glycerol-salt composite hydrogel fibers.^75^ The precursor solution was injected into a silicone tube (diameter: 1 mm) and then subjected to UV irradiation (365 nm, 10 W) for 30 min to obtain hydrogel fibers and the original hydrogel fibers were further stretched to create adhesive hydrogel fibers of different diameters (Figures 9A and 9B). It was demonstrated that the hydrogel fibers were able to adhere to various materials and maintain adhesion during stretching. The reversible adhesion stress of the hydrogel fibers to the polypropylene plates was 1.42 kPa and to the glass was 1.96 kPa. It showed good durability, repeatability and stability when used as strain sensors.

### 3D printing

**Figure 10:**
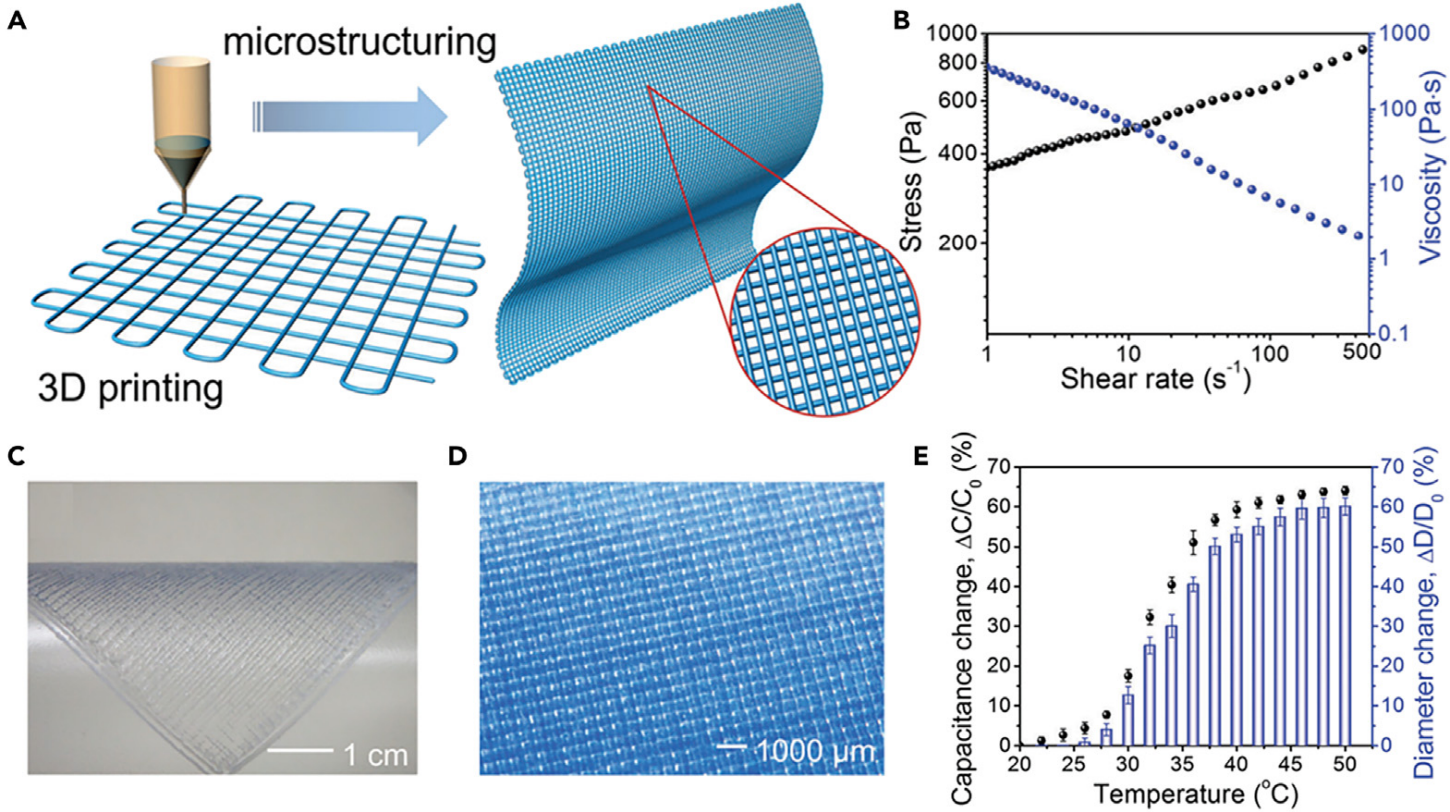
Hydrogel fibers prepared by 3D printing (A) Schematic illustration of the 3D printing process and the grid microstructure of a printed hydrogel film. (B) Viscometry results of the hydrogel. (C) A photo of the printed hydrogel film. (D) A photo of the grid-structured hydrogel. (E) The capacitance-temperature and diameter-temperature relationships of the thermo-responsive ionic skin. (A–E) Reproduced with permission.^79^ Copyright 2017, The Royal Society of Chemistry.

3D printing is a promising additive manufacturing technology with the advantages of rapid prototyping and customizability.^76^ 3D printers commonly work in laser-assisted printing, extrusion printing, and inkjet printing.^77^ Among them, the most common method to prepare hydrogel fibers is extrusion printing. Zheng et al. fabricated 3D hydrogel fibers by extruding highly viscoelastic poly(acrylic acid-coacrylamide) (P(AAc-co-AAm)), poly(acrylic acid-co-N-isopropyl acrylamide) (P(AAc-co-NIPAm)) solutions and their mixtures using multiple nozzles, and then the structure is gelled in FeCl_3_ solution to obtain 3D gel structure.^78^ The hydrogel fibers had uniform diameters (∼1 mm) and clear boundaries, and the hydrogel mesh (spacing: 1.5 mm) was created by orthogonal stacking. P(AAc-co-AAm) hydrogel fibers have mechanical properties comparable to those of bulk hydrogels, with a tensile strength of 2.38 MPa and an elongation of 802%. By integrating responsive and non-responsive gel fibers, fast and ordered deformation of macroscopic 3D layered materials was achieved. Lei’s group prepared thermally responsive hydrogel fiber membranes with grid structure by 3D printing, as shown in Figure 10A.^79^ Hydrogels were prepared by micelle co-polymerization of N, N-dimethylacrylamide (DMA) and n-octadecylacrylate (C18) in aqueous NaCl solution containing sodium dodecyl sulfate (SDS). The hydrogels were loaded into an extrusion tube with a temperature controller, and after melting at 45°C, the homogeneous and transparent hydrogel was extruded by 3D printing using a needle (diameter: 0.41 mm). Figure 10B depicts the viscosity and stress as a function of shear rate. The results showed that the hydrogel had shear thinning property, which is applicable to 3D printing to reconstruct the hydrogel structure. The photo of the printed hydrogel film is shown in Figures 10C and 10D, with a grid structure. The stimulus-responsive hydrogel was incorporated into the capacitor circuit to form ionic skins, which can be used as a wearable thermal sensor (Figure 10E).

### Microfluidic spinning

**Figure 11:**
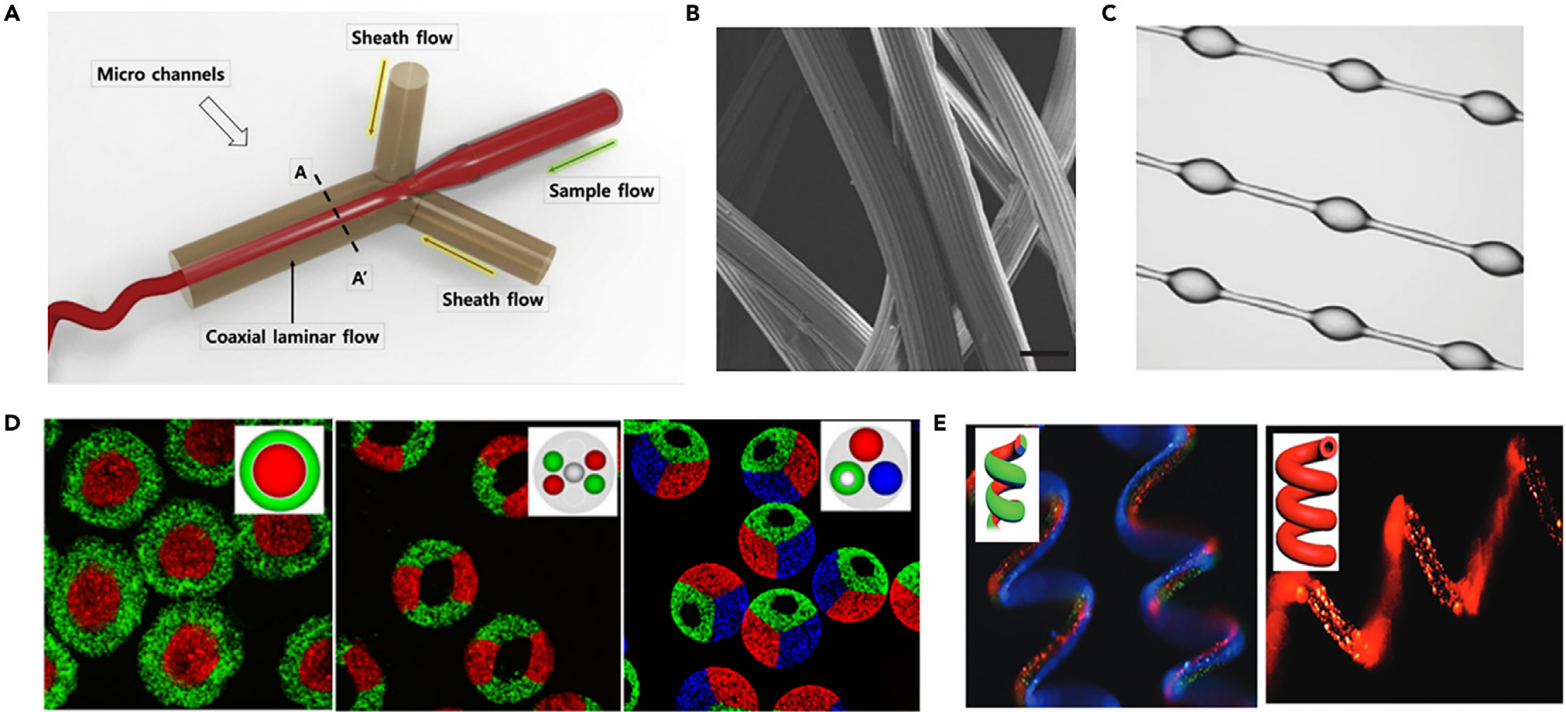
Hydrogel fibers with different morphological characteristics prepared by microfluidic spinning (A) Schematic of microfluidic spinning. Reproduced with permission.^145^ Copyright 2016, Elsevier Ltd. (B) Grooved fiber. Reproduced with permission.^146^ Copyright 2011, Macmillan Publishers Limited. (C) Knotted fiber. Reproduced with permission.^147^ Copyright 2016, Wiley-VCH. (D) Heterogeneous fiber. Reproduced with permission.^148^^,^^149^ Copyright 2014, WILEY-VCH. (E) Helical fiber. Reproduced with permission.^150^ Copyright 2017, WILEY-VCH.

In addition to these common methods for preparing hydrogel fibers, there are some novel spinning methods such as microfluidic spinning. Microfluidic spinning is a technology that combines pre-polymerized fluid propulsion and receiver traction with simultaneous formation of a cross-linked network, which focuses on the precise integration of multiphase laminar flow and regulation of the state of each phase in the microchannel environment, leading to the preparation of micro/nanoscale hydrogel fibers with controllable size and morphology (Figure 11A).^80^^,^^81^ By designing microfluidic chips to regulate the shape of the laminar flow in the microchannels, fibers with different morphological characteristics (e.g., tubular, grooved, helical, knotted) can be prepared (Figures 11B–11E).^82^^,^^83^^,^^84^^,^^85^^,^^86^^,^^87^ The hydrogel fiber crosslinking process includes photopolymerization crosslinking, chemical crosslinking and ionic crosslinking.

**Figure 12:**
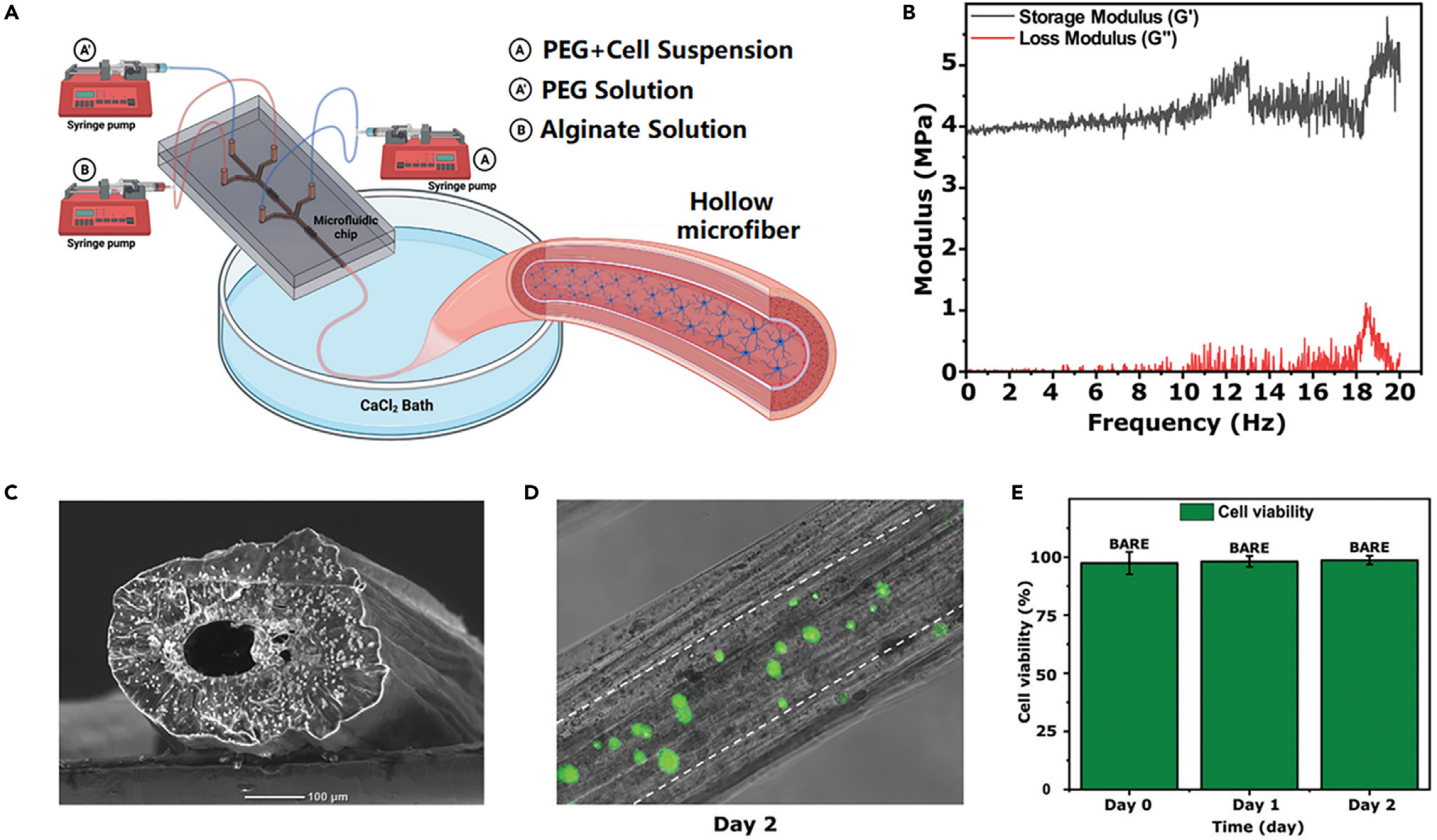
Hydrogel fibers prepared by microfluidic spinning (A) Schematic of the microfluidic fiber fabrication. (B) Frequency sweep test performed on the hydrogel fibers. (C) The cross-sectional SEM image of the fiber. (D) Live/dead cell assay images (Day 2) of the cells seeded on the inner surface of the alginate based hollow microfibers. (E) Bar graph of the cell viability from Day 0 to Day 2 for cells seeded in the hollow microfibers. (A–E) Reproduced with permission.^89^ Copyright 2022, the Authors.

Bow-tie shaped fibers with controlled size and shape were fabricated from poly(ethylene glycol) diacrylate hydrogels under a microfluidic system and a photopolymerization strategy (UV intensity: 153 mW).^88^ The shear force at the interface between the two fluids was varied by using different sheath core flow ratios to adjust the size of the hydrogel fibers. Fibers with controlled structures were fabricated by embedding four chevrons in the microchannel design to vary the lateral and vertical hydrodynamic focusing forces. When the core flow rate was 60 μL/min and the sheath flow rate was kept constant at 100 μL/min, the strain and stress at break were 39.7% and 5.9 MPa, respectively, and the mechanical properties of fibers changed when the core flow rate was different. In 2022, they designed a microfluidic-based method for cell inoculation within alginate hollow microfibers (Figure 12A).^89^ The storage modulus (*G*′) was comparatively higher than the loss modulus (*G*″) in the frequency range 0–20 Hz, indicating high elastic behavior of the hydrogel fibers (Figure 12B). Hollow hydrogel fibers with variable diameter can be fabricated by microfluidic spinning method, and its SEM image is shown in Figure 12C. Figure 12D shows the live/dead fluorescence image of the mouse astrocyte cells that were seeded on the inner surface of the nonadditive-based hollow microfibers, and an increasing trend in the cell viability is observed between Day 0 and Day 2 (Figure 12E).

## Device applications

Generally, hydrogels show an excellent responsibility to a wide range of environmental stimuli through volumetric phase changes of the network, changes of shape/size, and/or changes of optical and electrical properties. Thus, hydrogels are widely reported as flexible sensors and actuators. In addition, the composition, shape, and structure of hydrogel fibers can be easily modified to meet the needs of different application scenarios. In this section, we will briefly introduce the preparation methods and several popular applications of hydrogel fibers, with emphasis on sensor applications.

### Strain sensor

The requirements of CHFs as wearable sensors are not only to be able to deform with the movement of the human body, but also to have certain toughness, stretchability, flexibility and comfort. External force exerted on the CHFs leads to the changes in their electrical properties, thus they are able to detect the magnitude, direction and frequency of the external stress.^90^ These characteristics put forward high expectations for the materials selection, structural design and fabrication of hydrogel fibers.

**Figure 13:**
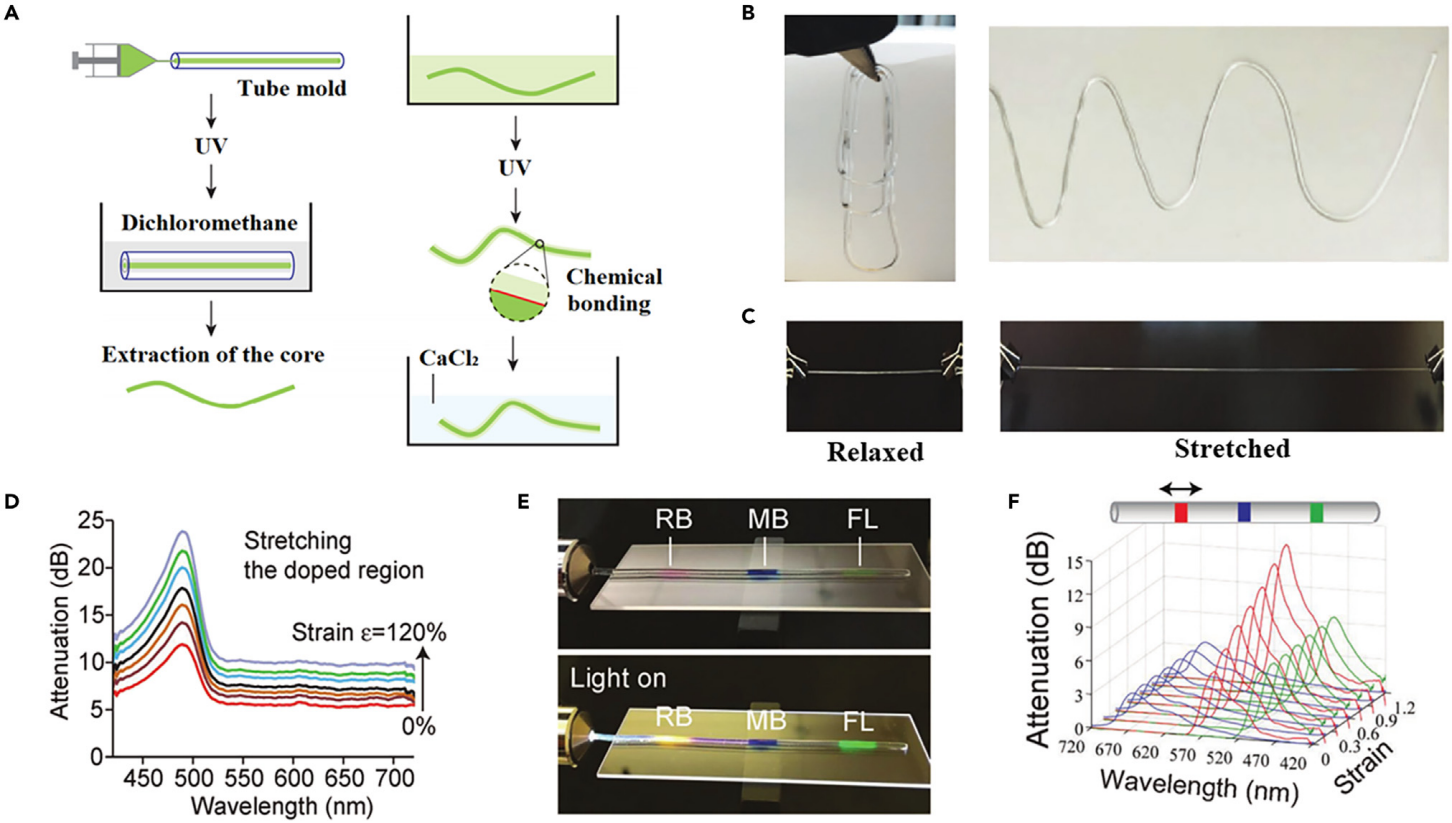
Strain sensors based on core/shell structure hydrogel fibers (A) Fabrication steps of a core-clad tough hydrogel fiber. (B) Photos of fabricated hydrogel fibers. (C) Photos demonstrating 3 times stretching of a hydrogel fiber. (D) The attenuation spectrum measured when the sensing area was stretched. (E) Photos showing a dye-doped fiber on a glass slide, without (top) and with (bottom) excitation broadband light. (F) Extracted absorption spectra of the sensor (red) when local strain was applied to it. (A–F) Reproduced with permission.^91^ Copyright 2016, WILEY-VCH.

Typically, CHFs cannot simultaneously achieve both high electrical conductivity and excellent tensile properties. The conductivity decreases with increasing strain and it is almost impossible to maintain electrical conductivity when the strain exerted on CHFs exceeds 1000%. Guo et al. reported a highly tensile hydrogel fiber material with a core/shell structure using sodium alginate and PAAm as raw materials, as shown in Figure 13A.^91^ The precursor for the core was synthesized by mixing aqueous solutions of acrylamide, 2 wt % sodium alginate, N, N-methylenebisacrylamide, and ammonium persulphate with calcium sulfate (CaSO_4_) and N, N, N′, N′-tetramethylethylenediamine (TEMED). Precursors were injected into a silicone tube mold using a syringe and cross-linked in nitrogen atmosphere under UV light at 50°C for 30 min. The core hydrogels were extracted and impregnated with sodium alginate-PAAm precursors in MES buffer (pH 6.0), and the clad-coated core fibers were cured by UV irradiation (Figure 13B). A new strain sensing principle was demonstrated using the unique physical and optical properties of optical fibers, where organic dyes were mixed into the porous matrix of the hydrogel by solution doping to achieve distributed strain sensing with a large dynamic strain range of 120%. The hydrogel fibers can be stretched up to 700% and then relaxed over multiple cycles (Figure 13C). The attenuation of light increases with the tensile strength when the hydrogel fiber is stretched with the addition of dye, and the change is larger than that of the undoped fiber (Figure 13D). Therefore, such hydrogel fibers can be used as strain sensors, and when the external object to which the hydrogel is affixed undergoes deformation, the specifics of the deformation can be reflected by observing the change in the color shade of the hydrogel fiber (Figures 13E and 13F). In 2019, Yin et al. proposed a core/sheath structure hydrogel fiber coated with a dielectric layer that can be stretched to more than 2000% of its initial length.^92^ The template method was used to fabricate the PAAm hydrogel fiber in a capillary tube. The photosensitive polyimide (PSPI) polymer was then dissolved in toluene, and the bare hydrogel fiber was dipped into the mixture, which was obtained after the self-volatilization of toluene. When stretched to 1300% of the original length, the fiber is still able to conduct electricity. Although it is not as conductive (0.16 S m^−1^) as fibers doped with carbon nanotubes or graphene, it has strong tensile properties. The hydrogel fibers are attached to the clothes and then fixed to the knee to detect squatting movements by applying alternating voltage to the fibers. The movement range and speed of the human body can be obtained from the change of electric current.

**Figure 14:**
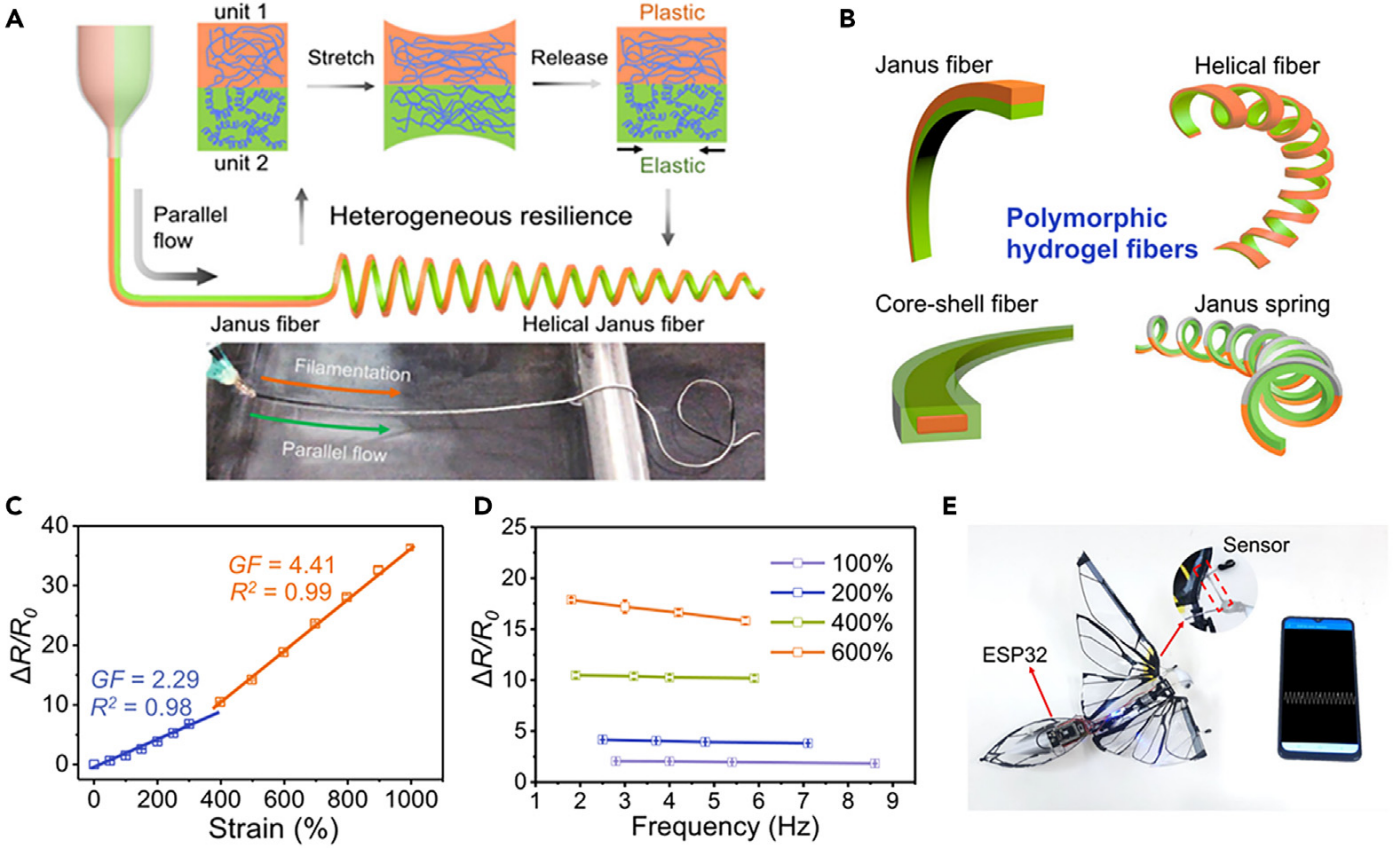
Strain sensors based on polymorphic hydrogel fibers (A) Schematic illustration of layered, monolithic hydrogels of varied macromolecule conformations. (B) Polymorphic hydrogel fibers based on conformation formation of macromolecules. (C) Resistance changes of the hydrogel fiber versus tensile strain, and the gauge factor (GF). (D) Resistance changes versus stretching frequency at different strains. (E) Photograph of the robotic bird installed with the hydrogel fiber sensor, bluetooth chip and power source. Reproduced under the terms of the Creative Commons Attribution License (http://creativecommons.org/licenses/by/4.0/).^22^ Copyright 2022.

CHFs with large stretchability was reported by introducing a macromolecular conformational shaping strategy that enables mechanical programming of devices based on polymorphic hydrogel fibers (Figure 14A).^22^ The prepared microfibers can take the form of built-in layering to generate easily customizable shapes of hydrogel fiber devices, such as fibers/ribbons, Janus fibers, multilayered fibers, core-shell fibers and helical fibers (Figure 14B). Hydrogel fibers can be programmed to have adjustable mechanical properties, from soft to ultrahigh rigid, brittle to ultra-stretch. The prepared fibers enabling the extraordinary applications of hydrogel electronics, namely high-strain (1000%) and ultra-fast response (∼30 ms) fiber sensors in robotic birds (Figure 14C). The sensing frequency can reach up to 5.7 Hz as measured, even at 600% strain (Figure 14D). Figure 14E shows the application of the hydrogel fiber as a sensor for wirelessly monitoring the wing beating movement of a robotic bird.

**Figure 15:**
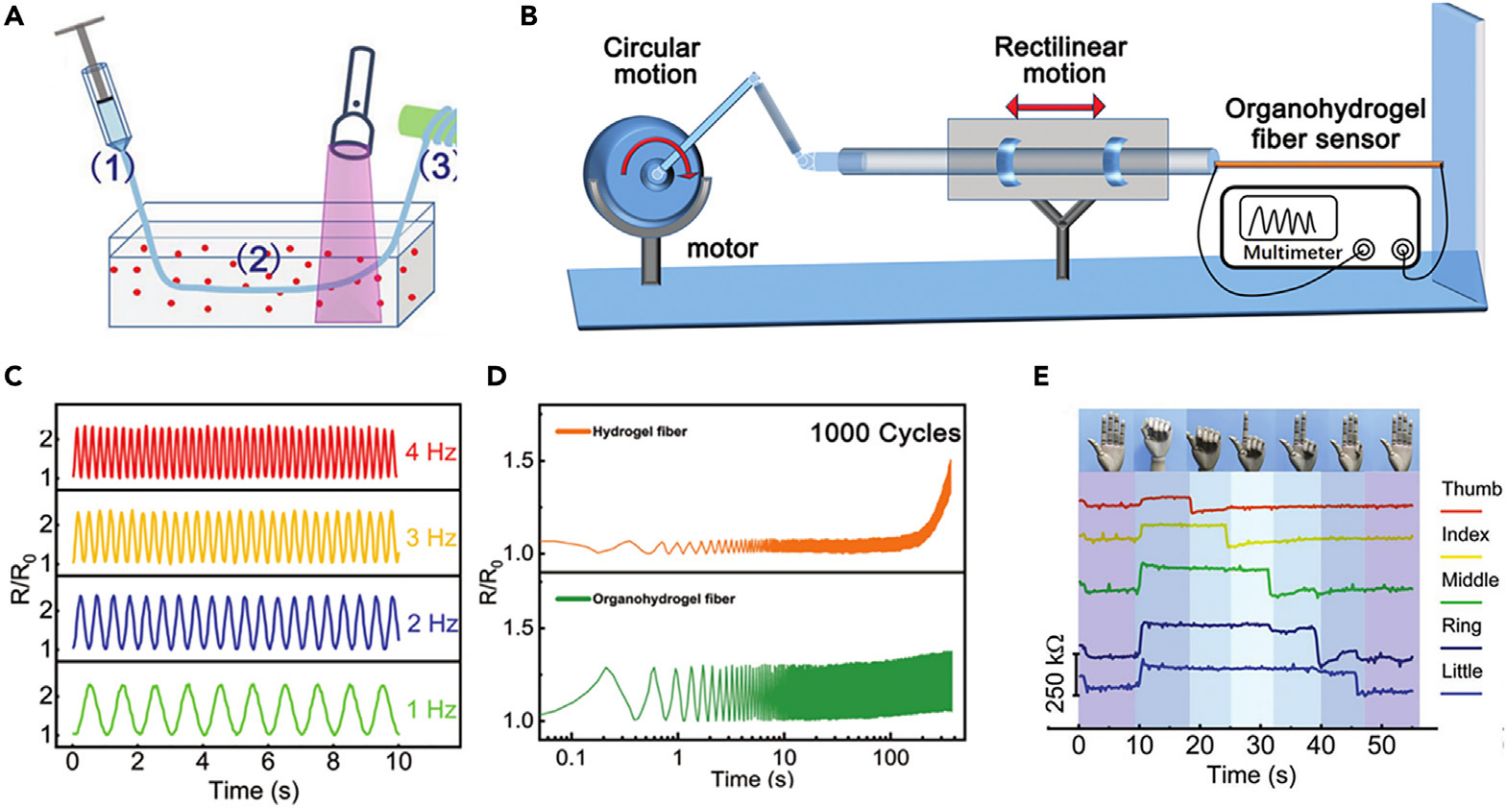
Strain sensors based on anti-freezing and water-retention hydrogel fibers (A) Schematic of the wet-spinning process of hydrogel fibers. (B) Schematic of the customized equipment used to simulate the motion of an engine valve. (C) Resistance changes of hydrogel fiber strain sensor at different stretching frequencies up to 4 Hz. (D) Distinct response of resistance (*R*/*R*_0_) of organo-hydrogel fiber and hydrogel fiber during cyclic stretching for 1000 cycles. (E) Real-time response of a five-channel data glove system made of organo-hydrogel fibers to finger flexion and extension. (A–E) Reproduced with permission.^93^ Copyright 2020, WILEY-VCH.

The characteristics of hydrogel with high water content also cause some issues in practical applications, such as the dehydration in ambient and freeze below 0°C. To extend the application range of conductive hydrogel fibers and to be able to endure in extreme environments, hydrogel fiber sensors need to have anti-freezing and water retention properties. A binary solvent consisting of glycerol and water can keep hydrogel fibers stable at low temperatures or in dry environments. Song et al. produced hydrogel fibers by wet spinning using sodium alginate (SA) and polyethylene glycol diacrylate (PEGDA), and then immersed the prepared hydrogels in a mixture of glycerol: water (1:1) with CaCl_2_ and KCl (Figure 15A).^93^ The organic hydrogel fibers exhibited excellent frost protection (<−80°C), transparency, and stretchability, and keep stable for a long period of five months. They customized a device to simulate the engine valves (Figure 15B) and the hydrogel fiber sensor was placed between the moving valve and a fixed board. The sensing function is achieved by measuring the change in resistance of hydrogel fiber, which is capable of accurately capturing movements with a frequency of 4 Hz and a velocity of 24 cm s^−1^, and can be used to detect fast cyclic movements, as shown in Figures 15C and 15D. The gauge factor (GF) of the organic hydrogel fiber sensor is 1.04 at 0–50% strain and 1.87 at 50–200% strain. Hydrogel fibers can be woven with common fabrics to fabricate a wristband-based strain sensor to detect strain in different directions of the finger (Figure 15E). In addition to the preparation of wearable anisotropic sensors, hydrogel fibers can be used as electrodes to obtain electrocardiograms, which have promising applications in the biomedical field. Using template method, PEDOT:PSS/PVA hydrogel fibers with a diameter of 24 μm were prepared by Shi et al. with glycerol as moisturizer.^73^ Two fine copper wires were used as leads and connected to both ends of the fiber by conductive silver glue to prepare the sensor. The other end of the copper wire that was not in contact with the hydrogel fiber was connected to the test instrument. The prepared sensors have excellent flexibility and mechanical strength, with an ultimate tensile strength of 13.76 MPa when the elongation at break is 519.9%. In addition, the hydrogel fibers have outstanding water retention properties and low temperature resistance because of the addition of glycerol. The sensor can still work flexibly after being placed in an atmospheric environment for 1 year. The sensor can be used under the harsh environment, and withstands 1000 repetitions of stretching and shrinking at a low temperature of −60°C stably. The sensor had good response in the detection range from 0.01% strain to 130% strain, with the gauge factor increasing from 0.5 to 5.4, indicating that the sensor can be used for monitoring complex human movements such as joint flexion, pulse, swallowing, etc.

CHFs based on conductive polymers were fabricated to enhance the sensing properties. For instance, Wu et al. used a combination of *in situ* oxidative polymerization to prepare sodium alginate/polyaniline/graphene oxide (SA/PANI/rGO) hydrogel fibers by wet spinning method using calcium chloride solution (5 wt %) as coagulation bath.^94^ The diameter of the prepared SA/PANI/rGO hydrogel fiber was 0.18 mm and the hydrogel fiber had excellent flexibility with a maximum strength of 10.4 MPa and an elongation of 154%, strong water absorption capacity (11.4 g/g), and desirable conductivity (0.5 S/m). The SA/PANI/rGO hydrogel fiber was installed on various body parts to provide real-time sensing of body movements by detecting resistance changes. As strain sensors, the hydrogel fibers showed sensitivity and repeatability in six tensile release cycles at different strains (10%, 20%, and 50%) and could be used to monitor palm, elbow, and knee movements. The gauge factor ((*R*− *R*_0_)/*R*_0_) of the hydrogel fiber sensor ranged from 0.14 to 1.92, which indicates that the hydrogel sensor has ideal sensitivity. Chen et al. also fabricated conductive hydrogels from polyaniline. First, polyaniline fibers were prepared by electrospinning and dispersed in an aqueous solution by a high-speed homogenizer.^95^ Then, konjac glucomannan (KC) powder, K-carrageenan (KGM) powder and LiCl were added to the solution, and the hydrogel was formed by stirring well. The hydrogels showed excellent mechanical properties (strength: 239.3 kPa, strain: 340.69%) and good electrical conductivity (0.73 S m^−1^) due to the contribution of polyaniline fibers and LiCl. The prepared hydrogels were able to accurately monitor the motion of body parts, including the index finger, elbow, wrist, and knee. However, this work only detected simple movements of the four body parts with joint flexion, and the strains were all relatively large; the response of the sensor at small strains, such as vocalizations and pulses, was not measured.

**Figure 16:**
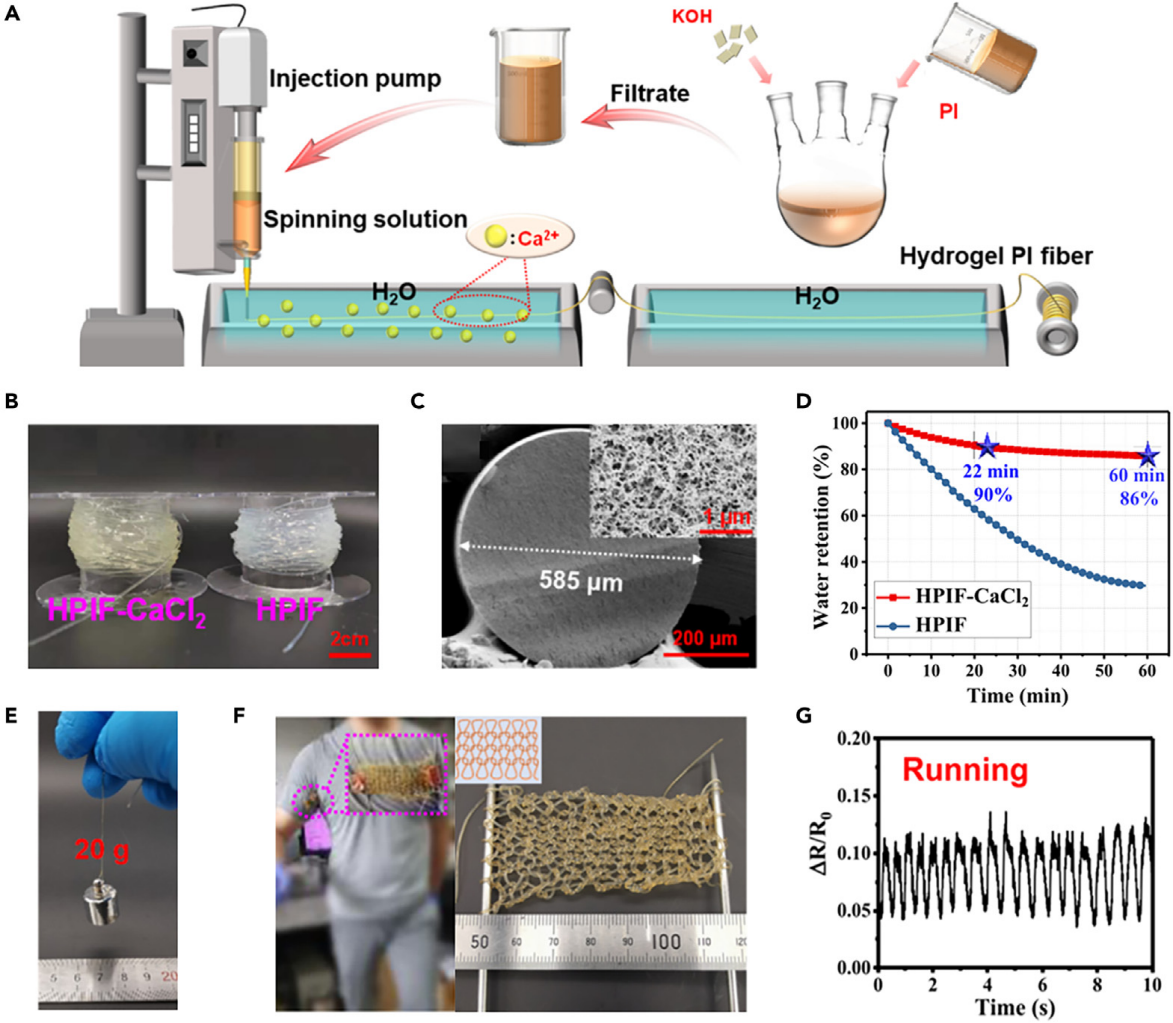
Textiles woven from hydrogel fibers that can be used as stress sensors (A) Schematic fabrication process of HPIF-CaCl_2_ fibers via wet spinning. (B) Digital photograph of the as-prepared HPIF and HPIF-CaCl_2_. (C) SEM images of freeze-dried HPIF-CaCl_2_. (D) Water retention−time curves of HPIF and HPIF-CaCl_2_. (E) Photograph of a single hydrogel fiber with a tensile weight of 20 g. (F) Photograph of a hand-knitted textile. (G) Movement processes monitored by hydrogel fiber fabric strain sensor. Reproduced with permission.^96^ Copyright 2021, American Chemical Society.

Hydrogel fiber sensors can be woven into two-dimensional structures on fabrics (e.g., sleeves) with excellent breathability, lightness, and stretchability for stable long-term monitoring of body signals. Li et al. prepared ionic polyimide (PI) hydrogel fibers by a wet spinning method. The PI salt/N-methylpyrrolidone (NMP) solution was filtered to remove the remaining KOH, and then the spinning solution was extruded from an injection needle with a diameter of 0.6 mm into the solidification bath (Figure 16A).^96^ The prepared ionic PI hydrogel fibers (HPIF) and HPIF-CaCl_2_ (diameter:585 μm) showed transparent and buffy appearance, respectively, as shown in Figures 16B and 16C. The resulting hydrogel fibers showed excellent electrical conductivity (∼2.1 S m^−1^), high water retention (Figure 16D) and had remarkable mechanical properties with a tensile strength of 2.5 MPa and elongation at break of 215%. As displayed in Figure 16E, A single HPIF-CaCl_2_ hydrogel fiber can withstand 20 g tensile load without breaking. The prepared fibers are strong and flexible and can be woven into monolithic fabrics that act as strain sensors to monitor the activity of body parts during exercise, such as running, jogging, walking, and resting (Figures 16F and 16G). Wang et al. prepared transparent conductive polymer fiber hydrogels, namely poly(polymerizable deep eutectic solvent (PDES)) fibers, using the template method of photopolymerization.^97^ The fibers exhibited excellent stability in dry environments at high (100°C) and low temperatures (−30°C), high water retention, excellent mechanical properties (maximum strength = 500 kPa, elongation = 300%) and sensing properties. In addition, the poly (PDES) fiber is woven into a common structured sensor on fabric with breathability and high damage resistance, which is capable of monitoring human arm’s stretching, bending and rotating stably and accurately.

### Temperature sensor

Human skin not only senses mechanical stimuli such as strain and pressure, but also has the ability to sense temperature changes in the environment, so temperature sensing is also an essential aspect of sensor applications. Temperature-responsive materials are more frequently used, and the temperature point at which the response is observed is called the critical temperature (*T*_c_).

**Figure 17:**
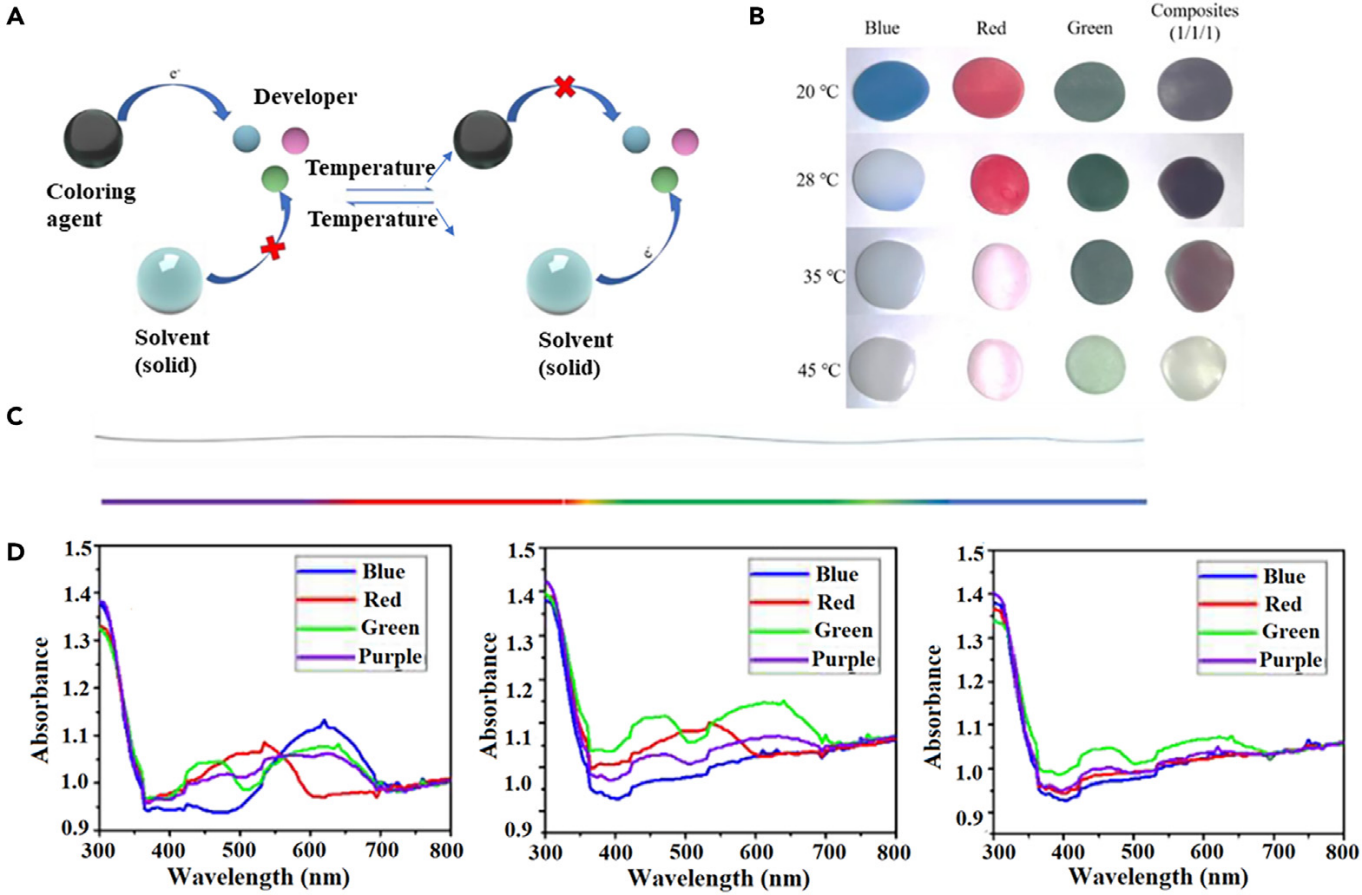
CHFs-based temperature sensors (A) Reversible color development mechanism of temperature-sensitive particles. (B) Reversible thermochromic process of hydrogel at different temperatures of 28°C (blue), 35°C (red) and 45°C (green). (C) Different temperature-sensitive particles are injected to form organic hydrogel fibers with gradient colors. (D) The UV-Vis spectrum of the organic hydrogel with different color temperature-sensitive particles at 20°C, 28°C and 35°C. (A–D) Reproduced with permission.^98^ Copyright 2022, Elsevier B.V.

In 2022, Wang's group prepared hydrogel fibers using hydroxyethyl cellulose (HEC) and polyvinyl alcohol (PVA) by template method.^98^ The PVA-HEC hydrogel fiber was immersed in an aqueous sodium chloride-glycerol solution to prepare organo-hydrogels with high strength and high electrical conductivity. To make the organic hydrogels temperature observable, temperature-sensitive particles were added to the organic hydrogels and squeezed into polyvinyl chloride (PVC) tubes to prepare the fibers. The reversible color development mechanism of the temperature-sensitive particles is shown in Figure 17A, and the thermochromic temperatures of the blue, red and green temperature-sensitive particles are 28°C, 35°C and 45°C, respectively (Figure 17B), and these three particles were compounded to prepare multi-stage temperature-responsive organic hydrogel fibers (Figure 17C). When the temperature was increased to different temperatures, the corresponding peaks disappeared and the color faded (Figure 17D). The discoloration of the hydrogel was still obvious after the warming and cooling cycle tests of the hydrogel, indicating the stability of the hydrogel fiber as a temperature sensor. In this way, observable monitoring of multi-level temperature can be achieved. The temperature sensor allows a more visual observation of the temperature change, but the response time is not measured and has significant limitations in practical applications.

Conductive polymers can also be incorporated into CHFs. Ismail et al. prepared hydrogel microfibers by wet spinning technique using chitosan solution as raw material, and then prepared chitosan/polypyrrole hybrid microfibers by *in situ* chemical polymerization of pyrrole in aqueous solution.^99^ The electrical conductivity of the fiber was 310 S m^−1^, and the electroactivity was imparted by polypyrrole. With the gradual increase of temperature, the peak anodic potential decreased and the cathodic potential gradually increased. The chrono potential response of the microfibers at different temperatures as a function of the electrical energy consumed suggests that the hydrogel fibers have temperature sensing capability.

### pH sensor

pH monitoring is widely used in biomedical applications, such as wound healing and drug release. In the case of anionic networks, if ionization occurs (higher ambient pH), the hydrogel response corresponds to a higher hydrophilicity of the network and electrostatic repulsion between chains. Conversely, in the case where protonation occurs (lower pH of the solution), the network acquires hydrophobic behavior and a higher structural dense state. pH-sensitive hydrogels are able to produce reversible changes in volume, mass and elastic modulus with pH.

Yu et al. prepared new hydrogel fibers by blending hydrolyzed polyacrylonitrile (H-PAN) and soy protein (SP) in a laboratory scale wet spinning device.^100^ With increasing SP content, the hydrogel fibers exhibited excellent reversible pH-sensitive behavior, that is, they swelled in alkaline solutions and collapsed in acidic solutions, and were reproducible. Tamayol et al. used a microfluidic spinning method to prepare hydrogel microfibers containing pH-responsive dyes that change color according to the pH of the wound and can monitor wound healing.^101^ pH-responsive dyes were loaded into mesoporous particles and the resulting pH-responsive microfibers were flexible and can form good contact with the skin. The pH of the skin is slightly acidic, varying in the range of 4–6.6, and skin damage can raise the pH of the skin. Therefore, this pH-responsive hydrogel fiber holds promise for the preparation of highly porous oxygen-permeable wound dressings for monitoring chronic wound pH.

In 2020, Wang et al. prepared hydrogel microfibers and microtubules by photopolymerization of sodium alginate templates using a microfluidic device.^102^ Hydrogel monomer solutions containing N-isopropylacrylamide (NIPAm) and sodium acrylate (SA) or allylamine (AA) were irradiated with UV light to initiate *in situ* photopolymerization. Temperature and pH changes induce fully reversible and reproducible volume changes in microfibrils, so the fiber sensor can measure temperatures in the range of 25–38°C and pH in the range of 3–11. The higher the amount of SA or AA in the hydrogel, the greater the degree of change in microfibril diameter size on pH changes. Reversible volume changes in hydrogels are essential for controlling the rate of drug release. These hydrogel fibers will provide some ideas for the development of new pH hydrogel sensors.

### Humidity sensor

Hydrogel-based humidity sensors can monitor external humidity through the change of conductivity. The adsorption and desorption of water molecules leads to the variation of the electronic/ionic conductivity of hydrogels, so they have been demonstrated for humidity sensors.

**Figure 18:**
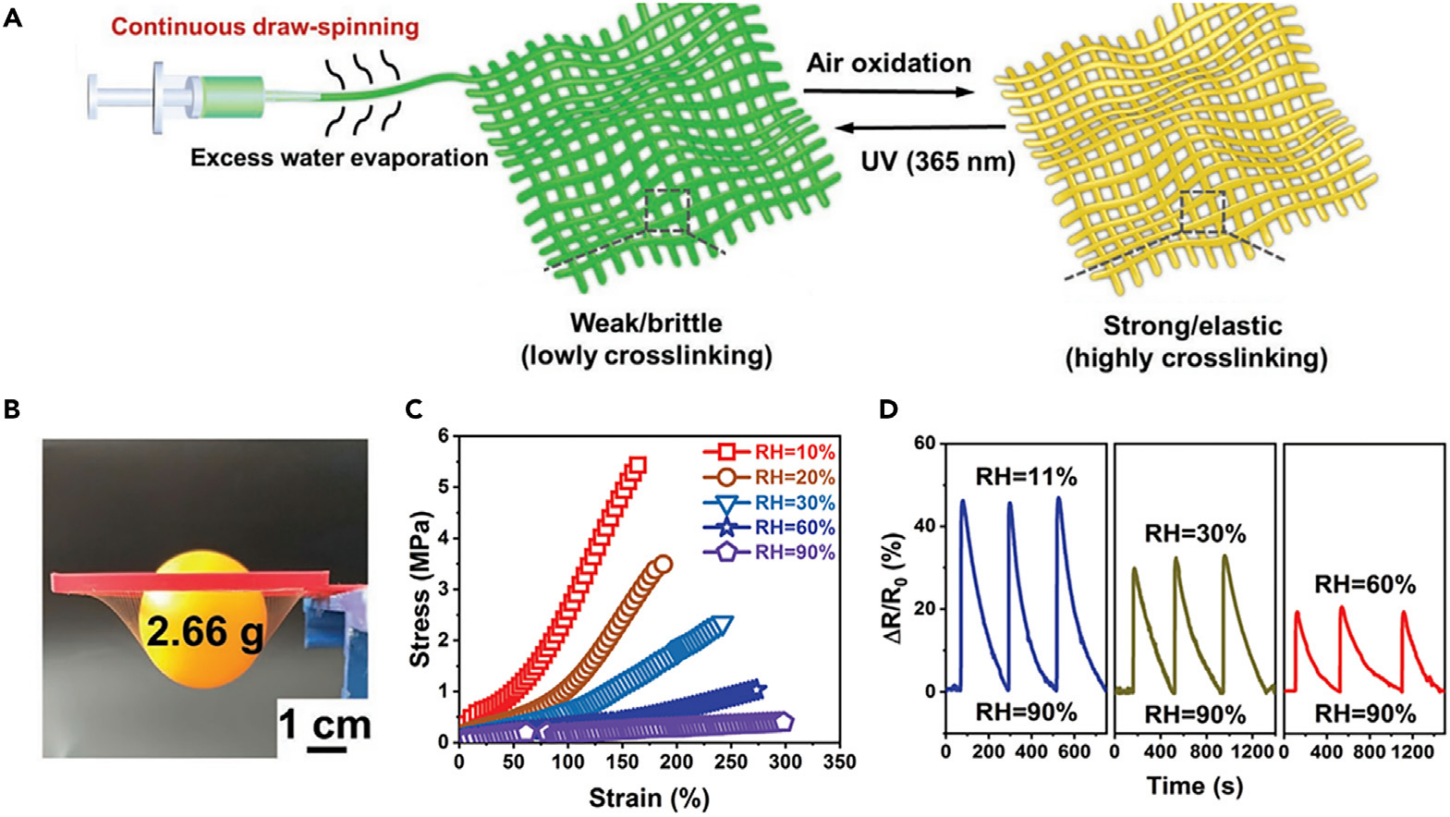
CHFs-based humidity sensors (A) Schematic fabrication of robust P(AAm-co-PAA)/Fe (III) hydrogel microfiber net. (B) The hydrogel fiber net is elastic and can withstand a table tennis ball. (C) Tensile curves of hydrogel fibers at different relative humidities. (D) Resistance changing curves as humidity switches between RH 90% and 11%, 30%, or 60%. (A–D) Reproduced with permission.^103^ Copyright 2020, WILEY-VCH.

For example, Ju et al. used a strategy of redox of iron citrate complexes for continuous draw-spinning of poly(acrylamide-sodium acrylate copolymer) hydrogel microfibers from aqueous/glycerol solutions.^103^ The hydrogel fiber was withdrawn directly from the syringe needle and immediately solidified with the evaporation of excess water (Figure 18A). This approach was able to modulate the fiber diameter to be electrically conductive (4.2 mS m^−1^), highly stretchable (Figure 18B) and homogeneous. The fiber can be stretched five times the original length with an elastic modulus of 0.27 MPa. In addition, the presence of iron citrate complexes and glycerol gives the fibers good freeze protection (−40°C) and water retention properties. The hydrogel fibers respond to light, humidity, and strain, allowing them to be highly sensitive to environmental changes. With the increase of humidity, the modulus and fracture stress of hydrogel fiber decreased significantly (Figure 18C). In particular, switching the humidity from 90% to 11%, 30%, or 60% leads to a significant increase in resistance, as the lower water content significantly reduces the ion migration rate (Figure 18D).

The variation of the optical property of CHFs was another indicator for humidity sensing. Gao et al. proposed an agarose hydrogel-filled photonic crystal fiber in-line interferometer for measuring relative humidity.^104^ The sensor is constructed by filling agarose hydrogel between an aligned single-mode fiber (SMF) and a photonic crystal fiber (PCF). Owing to the adjustable refractive index property of agarose gel, the diameter of the mode field of the propagated light varies with the external relative humidity. Relative humidity is measured by interrogating the fringe visibility of the white light interferogram. The experimental results show that he hydrogel fiber optic sensor has a humidity measurement range from 10%RH to 90%RH, and its sensitivity is up to 2.2 dB/RH.

In addition, hydrogel fibers are highly sensitive to humidity and moisture, making it possible to achieve humidity sensing.^105^ Liu’s group reported artificial spider silk produced by water evaporation-induced self-assembly of hydrogel fibers made of polyacrylic acid (PAA) and silica nanoparticles.^67^ The fiber showed a tensile strength of 895 MPa and a stretchability of 44.3%, achieving mechanical properties comparable to spider silk. A 5-cm-long PAA hydrogel fiber was supercontracted into a small ball with a diameter of 50 μm, meaning that the maximum supershrinkage could reach nearly 100%. Therefore, this artificial spider silk based on hydrogel fibers can be used in applications such as humidity sensors, wearable electronic devices, and artificial tendons.^106^

### Actuators

An actuator is a device that converts energy, which may be electric, heat, light, hydraulic, chemical, pneumatic, etc., to mechanical movement.^107^^,^^108^ Upon external stimuli, it would expand or contract in volume and shape, thus causing macroscopic mechanical deformation like bend, twist and grasp. The stimuli-responsive hydrogel actuator can realize a variety of complex movements such as bending, twisting and folding with large displacement through microfabrication technology.^109^ Hydrogel-based actuators are available in a variety of structures, including single-layer, bilayer, multilayer, and gradient structures, etc.^110^^,^^111^^,^^112^^,^^113^ Bilayer actuators are widely used because of their simple preparation and controllable/efficient deformability. Bilayer hydrogel actuators usually consist of two layers of hydrogels with different swelling rates, and the actuating layer can produce swelling or shrinkage behavior in response to environmental stimuli, whereas the other layer is a passive layer that does not respond to the same environmental stimuli, thus producing a change in shape to achieve the actuation effect.

**Figure 19:**
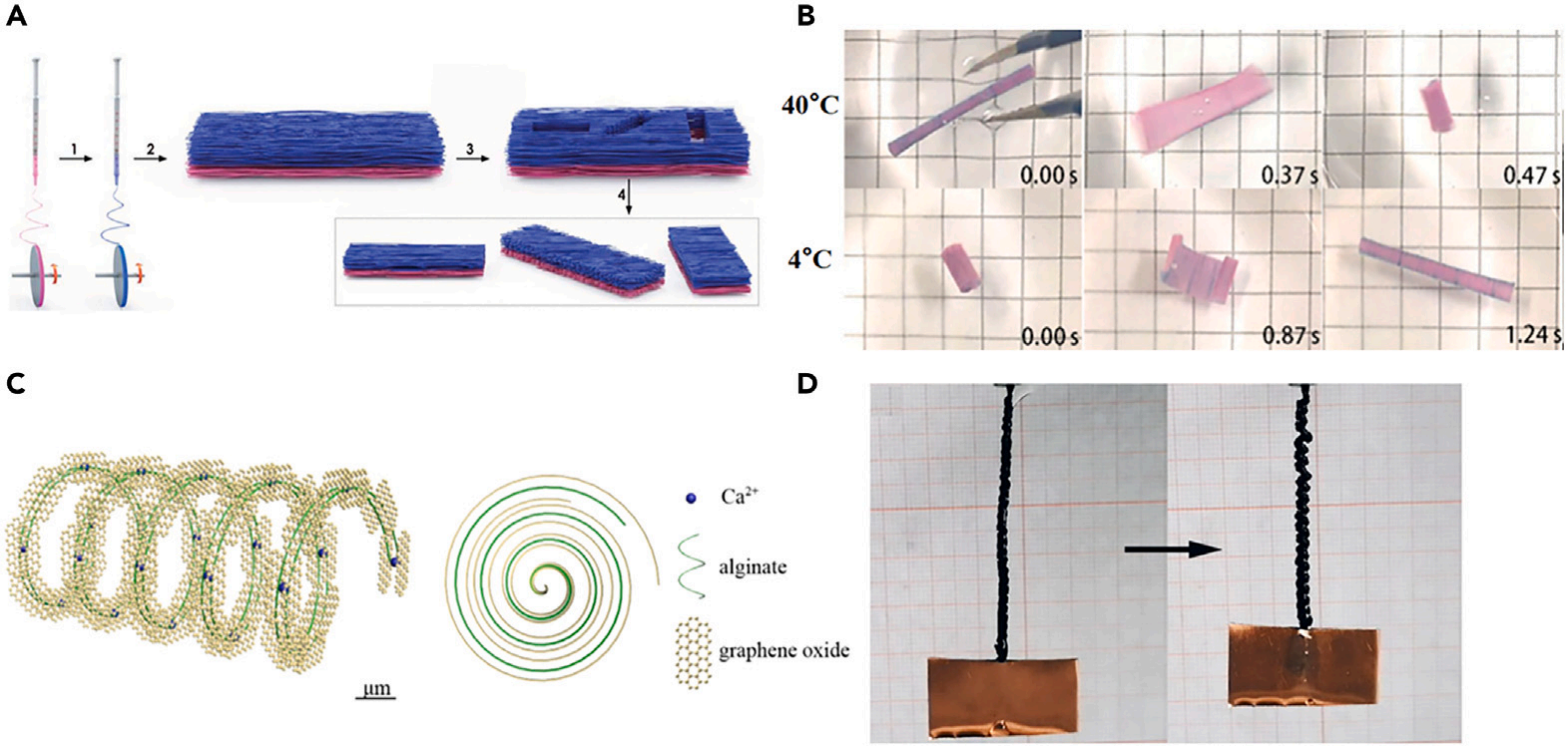
CHFs-based actuators (A) Actuator preparation process and related driving behavior of the fiber membrane. (B) Actuation time of the bilayer TPU/P(NIPAM-ABP) fibrous membrane in water at 40 and 4°C, respectively. (C) Schematic diagrams of helical structures of hydrogel fibers. (D) Application of helical GO/alginate fiber as a soft actuator in response to water adsorption. (A-B) Reproduced with permission.^114^ Copyright 2015, the authors, published by WILEY-VCH. (C–D) Reproduced with permission.^115^ Copyright 2020, American Chemical Society.

The bilayer structure hydrogel fiber membrane was prepared by Liu et al. via electrospinning method. This was the first thermosensitive hydrogel fiber membrane which obtains directional control of motion and fast response in first large size.^114^ The bilayer thermoplastic polyurethane (TPU)/poly (N-isopropyl acrylamide) (P(NIPAM-ABP)) fiber mat was made by electrospinning of two polymer solutions, spinning pure TPU first, followed by spinning of P (NIPAM-ABP) (Figure 19A). The TPU fiber membrane with oriented fibers acts as a passive layer in this bilayer membrane system, whereas the other fiber mat exhibits reversible anisotropic expansion/contraction in water relative to the dry spun fiber membrane, acting as an active layer. It is capable of reversible coiling, rolling, bending and twisting deformation in different controlled directions in more than 50 cycles, with surface and shape changes from the inside out (Figure 19B). Fiber actuators with a helical structure have also attracted extensive research, and An et al. used a combination of wet spinning and restricted drying to construct graphene oxide (GO)/sodium alginate hydrogel fibers with a helical structure (Figure 19C).^115^ After fixing the ends of the fibers, multi-step twisting was utilized during drying to construct hierarchically arranged helical structures. The tensile test shows that the hydrogel fibers can achieve the combination of high strength (∼2 MPa), high toughness (>5 MJ m^−3^) and large elongation (>700%). The authors investigated the driving properties of helical GO/alginate fibers for water adsorption, and the number of helices and the level of helical structure are important to produce large driving properties. The results showed that the helical structure can modulate the driving performance and helical fibers can be used as soft actuators (Figure 19D).

Hygroscopic twisting, shrinkage and tensile actuations was a general method to build actuator due to the hydrophilic properties of hydrogel. Liu et al. prepared hydrogel fibers by stretch-spinning method and coated carbon nanotubes on the surface to improve the water resistance of hydrogel fibers which exhibited twisting, shrinkage and tensile motions.^116^ Shrinkage or elongation was determined by the wrapping angle between carbon nanotubes and hydrogel fibers. If the carbon nanotube orientation direction is parallel to the hydrogel fiber direction, the actuator will contract and actuate in a humid environment, and if the wrapping angle is non-zero, the actuator will elongate and actuate. A fully reversible rotational degree of 1455° mm^−1^, contraction of 11%, and elongation of 54.8% were realized for torsional, contractile, and elongational actuators, respectively. The actuation work capacity for the contraction and the elongation can reach as high as 26.5 and 28.2 J kg^−1^. In 2022, they prepared spiral cotton hydrogel yarn (CHY) with nuclear shell structure by coating the cotton yarn with polyacrylic acid hydrogel.^117^ Once the relative humidity increased from 60% to 90%, the homochiral yarns showed 90% contraction, whereas the heterochiral yarns showed 450% expansion. The core-shell structure exhibits irreversible driving behavior in response to moisture, which is because of the super contraction behavior of the hydrogel fibers, making CHY a good candidate for fabricating fiber artificial muscles.

**Figure 20:**
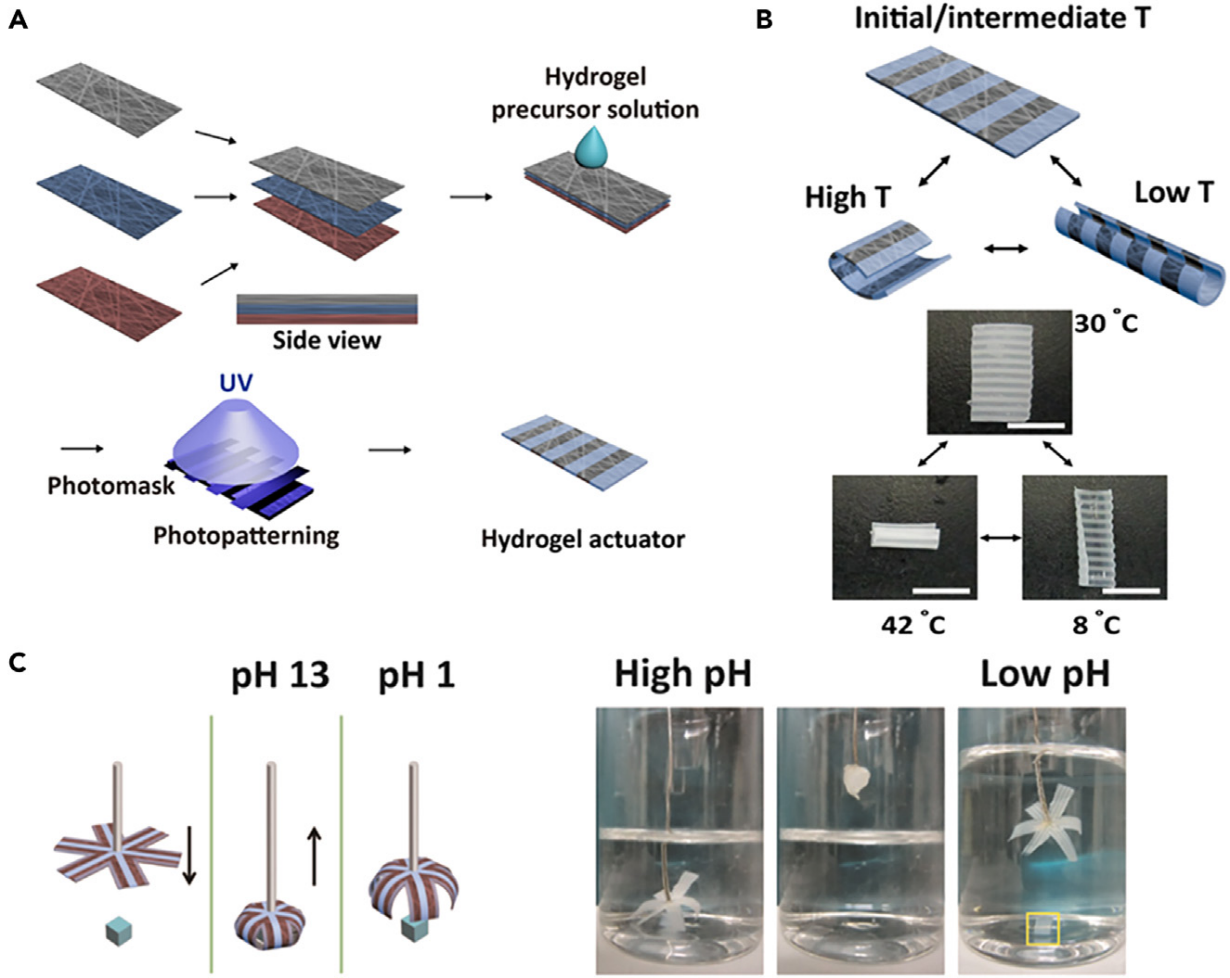
CHFs-based multi-response actuators (A) Schematic of the preparation process of multi-response soft actuators. (B) Temperature-responsive actuation of PNIPAAm/PCL-layered actuator. (C) “Grab-and-release” concept actuation. (A–C) Reproduced with permission.^118^ Copyright 2021, Elsevier B.V.

Electric field actuation capability was investigated by Zhao et al. PAMPS/PAM hydrogel fibers was prepared continuously by wet spinning method using poly(2-acrylamido-2-methylpropane sulfonic acid) (PAMPS) as lubricant.^31^ The complex actuation behavior of the hydrogel fibers, such as mimicking underwater biological movement or finger bending, was also regulated by electric field, providing a new strategy for the large-scale preparation of hydrogel fiber actuators. The actuator achieves maximum bending angles of 90°, 48°, 90° and 180° in the Mobula simulation, jellyfish simulation, 2-DOF simulation and hand simulation soft robot, respectively. This advantage is particularly significant for 2-DOF actuators, which in previous reports could only achieve maximum bending angles of 1.5°–28°. Cho et al. fabricated multi-responsive actuators in response to pH and temperature changes by combining electrospinning and hydrogel photolithography (Figure 20A).^118^ The soft actuator consists of stimulus-responsive hydrogel fibers as the active layer, non-responsive fibers as the passive layer, and micropatterned coupling hydrogel layer. In the case of the poly (N-isopropyl acrylamide) (PNIPAAm)/polycaprolactone (PCL) actuators, the bending response of the actuator can be observed by exposing the fabricated actuator to 8°C and 42°C deionized water (Figure 20B). A six-leaf flower-shaped PAA/PCL actuator was prepared and demonstrated to grasp and lift an object by bending drive at pH 13. The gripper was opened at pH 1, releasing the gripped object (Figure 20C).

## Conclusion and outlook

Hydrogel fibers show many unique properties because of their high specific surface area, high water content, mechanical durability and other structural advantages, which not only improve the material properties but also expand the application range. In this review, we summarize the recent advances in hydrogel fibers. The newly emerging requirement, characterization and fabrication of hydrogel fibers are comprehensively illustrated. In addition, device performance and applications of these hydrogel fibers based wearable sensors in human motion detection/monitoring and soft actuators are highlighted. Successful outcomes from these efforts shine a bright light on the potential applications of the hydrogel-based sensors in next-generation wearable electronic devices. However, a higher electrical conductivity, sensitivity and biocompatibility were not enough to implement these high-performance hydrogel-fiber-based soft electronics for practical applications, several imminent challenges need to be further investigated.

As the main component of hydrogel fibers is water, flexible electronic devices based on hydrogel fibers face a series of problems caused by dehydration. Compared to bulk hydrogels, hydrogel fibers have inferior water retention ability and loss ionic conductivity within several minutes without proper encapsulation, hindering their long-term applications. On the other hand, the hydrogel fibers will freeze and become brittle under sub-zero conditions, limiting their use in harsh environment.^119^ Thus, effective strategies to improve the water retention and anti-freeze ability of hydrogel fibers are highly demanded.

Second, the mechanical properties of the hydrogel fiber and the devices based on these materials are important for wearable and actuator applications. Hydrogels are typically soft and fragile materials, and one-dimensional hydrogel fibers are more delicate than bulk hydrogels, which may make them susceptible to damage or deformation during use. This may affect the performance and reliability of electronic devices. Researchers need to further explore ways to improve the mechanical durability of hydrogel fibers.^120^ The long-term stability and robustness of the devices at the real working conditions, where human sweating, body friction and environment temperature variation coexist, is largely unexplored. For wearable electronics that need to adhere to human tissue surfaces, common problems include slow adhesion formation, weak bonding, and low biocompatibility.^121^ Most hydrogels work well under dry conditions only, and their wet adhesion capability needs further validation.^122^ In addition to the pursuit of strong adhesion, the development of reversible adhesion is essential because of the urgent requirement for benign separation of electronic devices from adherent tissues. Reversible adhesion is crucial for repositioning misplaced bioadhesives and retrieval devices. Work in these areas would be important steps in moving flexible electronics based on hydrogel fiber materials toward future practical applications.

Third, hydrogel fibers show diverse applications in strain, temperature, pH and humidity sensing. But the development of soft electronics requires integration, efficiency and multidimensionality, unitary sensing ability is insufficient to meet future development. At present, most CHF-based sensors only show a single function, thus, how to improve the multifunctionalities of CHF-based sensors is a priority.^123^^,^^124^ New approaches need to be explored to improve the sensing capabilities of hydrogel fibers, such as by incorporating new materials, developing novel preparation methods or more sophisticated signal processing techniques that allow it to detect a wider range of parameters or provide more accurate and precise measurements. On the other hand, the integration of electronic units with different functions (including luminescence, energy harvesting devices, energy storage devices, smart sensors, data acquisition units and feedback systems) in one platform can effectively promote the development of flexible, wearable and portable electronic products.^125^ The variation of luminescence intensity of luminescent hydrogel fibers in response to external stimuli makes them promising for bioelectronics.^126^^,^^127^ For example, luminescent hydrogels allow continuous glucose monitoring because of the linear relationship between optical power and glucose concentration.^128^ Energy harvesting equipment can convert mechanical energy or heat energy around the human body into electricity, and integrate energy storage devices to build a self-powered system.^129^ Wearable items sewn with energy-harvesting hydrogel fibers, such as wristbands or sportswear, not only have self-lighting capabilities, but can also extract energy from human movement as an active sensor to detect human movement or temperature, and have applications in fields such as artificial intelligence and biomedicine.^130^ Moreover, the design concept of energy storage devices based on hydrogel fibers, such as supercapacitors, provides new power for the next generation of wearable and portable electronics.^131^^,^^132^

Fourth, hydrogel fibers are developing toward flexibility, but the response time of devices needs to be reduced significantly. Currently, the response time of CHF-based sensors to external stimuli ranges from tens of seconds to minutes, which is highly correlated with materials and device designs, where there is still much room left.

Fifth, hydrogel fiber actuators face similar problems as sensors.^133^ The common issues are slow response rate and low mechanical strength, which have been considered as the main obstacles hindering their further development.^134^ For instance, mammalian skeletal muscles have actuation power densities in excess of 10^4^ W m^−3^, a power density level that is ideal for artificial muscles used in medical devices and bionic robots. However, the power densities of existing hydrogel actuators are only in the range of 0.1 to 10^3^ W m^−3^, which is much lower than that of skeletal muscle.^135^ In addition, most applications of hydrogel fiber actuators are still in the conceptual stage, and long-term durablity must be resolved in practical applications.^136^ Therefore, the invention of new responsive hydrogels with fast response rate, high actuation power density and excellent mechanical properties represents a future direction of the field.^137^

Sixth, mass production in industry is still challenging because of the complex fabrication process, bare long-term stability and unitary sensing ability, leading to the application scenario of hydrogel fibers is still limited to laboratory conditions.^138^ Nowadays, optimization, structural design and multifunctional assembly rely on the interdisciplinary (e.g., textile, biological, mechanical and electronic) turns to promote hydrogel fibers into practicalization and industrialization. Moreover, although research on hydrogel fibers has developed rapidly in the past decade, for the materials/devices, device standardization, modeling, and simulation are required to understand the relationship between the performance of the pressure sensor and the structures (e.g., pore size/size distribution, dimensions, etc.) of the hydrogel fibers. This understanding may help develop materials with excellent sensing ability and multifunctionality.

Though with these challenges, there are several promising directions for future development of hydrogel fibers, such as wearable healthcare, biomedical, electronic skin, soft robotics, human-machine interactions and smart textiles. Hydrogel-based electrodes have been widely used for monitoring vital signs, the fiber topology extends their applications to smart drug delivery, wearable or implantable medical devices and soft robotics by combining drug-loaded microspheres, conductive materials and magnetic particles for controlled drug release, biosensing, and minimally invasive surgery.^139^ In addition, stretchable conductive hydrogel fibers are essential for the development of smart e-textiles. Incorporating hydrogel fibers can be incorporated into clothing or other textiles, giving the textiles a variety of functions that go far beyond traditional warmth/fashion functions, such as detection of environmental or body changes, energy generation and storage.^140^^,^^141^ This may lead to the development of clothing that can regulate temperature, monitor hydration levels, or provide feedback about posture and movement.
